# Construction and use of gene expression covariation matrix

**DOI:** 10.1186/1471-2105-10-214

**Published:** 2009-07-13

**Authors:** Jérôme Hennetin, Petri Pehkonen, Michel Bellis

**Affiliations:** 1 Centre de Recherches en Biochimie Macromoléculaire, CNRS, 1919 rte de Mende, 34293 Montpellier Cedex 05, France; 2 Laboratory of Functional Genomics and Bioinformatics, Department of Neurobiology, A.I. Virtanen Institute for Molecular Sciences, University of Kuopio, P.O.B. 1627, Kuopio 70211, Finland

## Abstract

**Background:**

One essential step in the massive analysis of transcriptomic profiles is the calculation of the correlation coefficient, a value used to select pairs of genes with similar or inverse transcriptional profiles across a large fraction of the biological conditions examined. Until now, the choice between the two available methods for calculating the coefficient has been dictated mainly by technological considerations. Specifically, in analyses based on double-channel techniques, researchers have been required to use covariation correlation, i.e. the correlation between gene expression changes measured between several pairs of biological conditions, expressed for example as fold-change. In contrast, in analyses of single-channel techniques scientists have been restricted to the use of coexpression correlation, i.e. correlation between gene expression levels. To our knowledge, nobody has ever examined the possible benefits of using covariation instead of coexpression in massive analyses of single channel microarray results.

**Results:**

We describe here how single-channel techniques can be treated like double-channel techniques and used to generate both gene expression changes and covariation measures. We also present a new method that allows the calculation of both positive and negative correlation coefficients between genes. First, we perform systematic comparisons between two given biological conditions and classify, for each comparison, genes as increased (I), decreased (D), or not changed (N). As a result, the original series of n gene expression level measures assigned to each gene is replaced by an ordered string of n(n-1)/2 symbols, e.g. IDDNNIDID....DNNNNNNID, with the length of the string corresponding to the number of comparisons. In a second step, positive and negative covariation matrices (CVM) are constructed by calculating statistically significant positive or negative correlation scores for any pair of genes by comparing their strings of symbols.

**Conclusion:**

This new method, applied to four different large data sets, has allowed us to construct distinct covariation matrices with similar properties. We have also developed a technique to translate these covariation networks into graphical 3D representations and found that the local assignation of the probe sets was conserved across the four chip set models used which encompass three different species (humans, mice, and rats). The application of adapted clustering methods succeeded in delineating six conserved functional regions that we characterized using Gene Ontology information.

## Background

Since the introduction of microarray technology in the 1990s, a large number of data sets have been produced in the field of transcriptome profiling and made publicly accessible through specialised repositories like the Gene Expression Omnibus (GEO) at NIH[1,2] or ArrayExpress at EBI [3,4]. Based upon the massive analysis of these types of data, a number of different approaches have been taken to develop integrated knowledge about the coexpression of genes [5], to search for regulatory elements in upstream regions of genes [6], to define transcriptional modules [7,8], to understand relationships between the interactome and transcriptome [9], and other applications.

The calculation of correlation coefficients between pairs of genes takes place at the very beginning of most studies involving the massive analysis of transcriptome microarray data sets, in particular those aimed at constructing transcription networks. The ultimate quality of these networks can therefore be influenced by numerous factors that are capable of affecting the quality of the correlation coefficients. Among these factors is the inter-laboratory reproducibility, which is of great importance because massive analysis requires collecting results originating in multiple laboratories.

Apparent discrepancies between the results of several independent transcriptomic studies in the same system, e.g. several types of mouse stem cells [10-12] or human embryonic stem cells [13], have highlighted the need for more research and for deeper analysis of the factors that control the accuracy and inter-laboratory reproducibility of microarray technology. In particular, large comparative studies addressing various technical points, involving several laboratories and comparing different platforms, have been conducted [14-18]. The interpretation of these results is difficult because of the large number of confounding effects that can bias any particular study (see [16]). Regarding the calculation of correlation coefficients, however, it has been possible to order these confounding effects according to their relative importance, revealing that the lab-effect is the most dominant. Indeed, significant differences can be observed between the results obtained when the same platform is used in different laboratories to study the same biological systems. When conducted in a realistic way, i.e. leaving each laboratory free to select the protocols used, all studies converge towards the conclusion that commercial oligonucleotide microarrays deliver results that are far more reproducible than those generated by cDNA microarrays. For example, in one study, a systematic evaluation of the relevance and biases of genetic networks extracted from different expression datasets showed that the biological relevance of networks constructed from Affymetrix data was remarkably higher than the relevance of gene networks inferred from cDNA data [19]. Since these two types of platform are comparable in terms of sensitivity, specificity, and accuracy when they are carefully handled in the same laboratory [20], we conclude that the standardization of the entire process, which is a characteristic of commercial oligonucleotide platforms, plays the main role in the reproducibility of the results by minimizing the lab effect. In contrast, cDNA platforms are strongly penalized by the absence of recommended protocols for the preparation of targets and the possibility of using different hybridization units, scanners, and software [14]. For these reasons, we have decided to focus our attention on data generated by commercial oligonucleotide platforms. Even more precisely, the Affymetrix platform appears to be optimal for analysis because concurrent platforms are poorly represented in the repositories.

Correlation values between genes may be obtained in two ways. Specifically, genes can be considered to be positively or negatively correlated either because their absolute expression levels follow a similar or inverse course across several biological conditions (referred to as coexpression), or because relative changes in their expression levels vary in the same or opposite direction in a series of comparisons between two biological conditions (referred to as covariation). Because individual signals obtained with double-channel platforms, such as spotted oligonucleotide or cDNA microarrays, cannot be used reliably, one must use variation measures (e.g. fold change) to calculate the correlations between genes. In contrast, with single-channel techniques such as in-situ oligonucleotide chips, no internal controls are necessary and the normalized signals can therefore be used to calculate gene expression correlations across several conditions. It follows then that the vast majority of massive analyses of transcriptomic results obtained to date with single-channel technique have only used coexpression.

One conclusion of analyses of inter-platform and inter-laboratory studies is that variation data are far more reproducible than expression level results (see, for example, the opposite conclusions reached by Kuo [21] and Irizarri [15] using expression level and log-fold change correlations, respectively). Using covariation instead of coexpression should therefore improve the quality of the correlation coefficients calculated from single channel results, which account for about 70% of the publicly available data [2].

While clustering techniques are often used to assemble substantial subsets of genes [7], when correlations are to be calculated on all possible pairs of genes most studies rely on the use of classical correlation measures such as Pearson's product-moment coefficient or Spearman's rank correlation coefficient. In contrast, other techniques like the mutual information score [22], normalized differences [23], or cosine correlation distances [24] are rarely used. To our knowledge, the suitability of the widespread use of these correlation measures for massive microarray analysis has not been questioned. The most obvious criticism we could make in this regard is that this type of statistical tool is too simplistic in view of the complexity of the relationships that exist between the transcript levels of any two genes observed across numerous biological conditions. Further, as the number of conditions under consideration rises, the probability increases that the two genes will have a positive correlation in one subset of conditions and a negative correlation in another subset. Depending on the relative importance of the two subsets, the correlation coefficient, calculated using either covariation or coexpression, will give a positive, negative, or non-significant value because all the conditions are analyzed together [25]. For example, there should be a positive correlation between a transcription factor and its targets. However, it is possible that, under a specific set of biological conditions, the expression of another isoform [26] of the factor or a post-transcriptional modification [27] will drastically change the factor's activity and give rise to a negative correlation with its targets (examples shown in [25] and [28]). This specific problem has long been recognized in the field of unsupervised machine learning, leading to the development of bi-clustering techniques [29]. However, nothing comparable has been developed to date for the calculation of gene correlations.

Another point to consider comes from inter-platform and inter-laboratory studies. The reproducibility of variation is sometimes assessed by considering the correspondence at the top (CAT), i.e., the percentage of changing genes in a test list that are also present in a reference list of the same size. More generally, methods comparing the most varying genes found in two independent studies are better able to support conclusions regarding inter-laboratory [15] or inter-platform consistency [13] than are methods relying on correlation coefficients.

Taken together, all of these results indicate that the popular methods that have been used to date for the massive analysis of results obtained in different laboratories with single channel technologies, which rely on correlation between expression levels, are not the best adapted and can be outperformed by methods comparing lists of genes showing statistically significant variations. We have developed such a method that allows the calculation of both positive and negative significant correlation scores using gene expression variations between pairs of biological conditions as data. When considering a given set of biological conditions, all studied using the same chipset model based on the single-channel technique, we first perform systematic comparisons between any two conditions (therefore, n conditions will give rise to N = n(n-1)/2 comparisons). For each comparison, genes are classified as increased (I), decreased (D), or not changed (N) by application of our Rank Difference Analysis of Microarray (RDAM) method ([30,31]). These multiple comparisons result in the assignation of an ordered string of N symbols to each gene, e.g. IDDNNIDID....DNNNNNNID. In the second step, two covariation matrices (CVM) are constructed by calculating statistically significant positive and negative correlation scores for any pair of genes by comparing their strings of symbols [32]. This new method, applied to four different large data sets encompassing three different species (Man, Mouse and Rat), allowed us to construct covariation matrices (CVMs) with similar properties. Further, we developed a technique to visualize, in three dimensions, the covariation networks encoded in these CVMs, and found that the local assignation of the probe sets was conserved across the four chipset models used. Finally, the application of adapted clustering methods allowed us to delineate six conserved functional regions that we characterized using Gene Ontology information.

## Results

### Data filtering

In this first section, we present the data that we used, explain how the data were organized, and describe briefly the quality filters that we developed. The filters were used to eliminate data that could potentially affect the construction of CVMs (a more comprehensive description of the entire data filtering process is available in the additional file 1).

The method that we used to calculate CVMs relies on our ability to perform systematic comparisons between any two biological conditions present in sets of experiments coming from different laboratories and on the availability of datasets obtained over a large panel of experimental conditions with the same standardized collection of probes. These two criteria, plus others described in the Introduction, led us to restrict our analysis to datasets originating from Affymetrix platforms, and to consider four chipset models in which the number of biological conditions studied was greater than 100 as of October 2004 (see additional file 2). We organized the downloaded data into a structure in which the samples are grouped according to the biological conditions and experiments. With this nomenclature, experiments and samples correspond, respectively, to Geo Data Sets (GDS) and Geo Samples (GSM) in GEO. A given biological condition groups all the replicated experimental points (Table 1, "before filtration" section).

**Table 1:** Platform origin of the data downloaded from GEO.

	**before filtration**			**first filtration**		**end filtration**		**NR filtration**	
chipset model	#exp	#biol	#point	#exp	#biol	#exp	#biol	#exp	#biol

Human Genome U95 Set	32	237	882	23	136	15	71	13	37
Human Genome U133 Set	24	174	538	19	137	18	126	13	88
Murine Genome U74 Version 2	56	351	808	50	251	37	205	36	89
Rat Genome U34 Set	22	183	543	18	119	13	96	13	41
	**134**	**945**	**2771**	**110**	**643**	**83**	**498**	**75**	**255**

For each model listed, the number of experiments (#exp), the number of biological conditions (#biol), and the number of samples (#points) are indicated. Columns labelled "before filtration" indicate the raw figures at the time the data were downloaded. Columns labelled "first filtration", "end filtration", and "NR filtration" indicate the figures obtained once the unusable (first and end) and redundant samples (NR) were eliminated. For these three groups, the number of samples is twice the number of biological conditions.

A preliminary step in the Rank Difference Analysis of Microarray (RDAM) [30,31] is the transformation of each signal into a relative rank, i.e. the placing of the signal value into the signal distribution, expressed on a continuous scale of 0–100. This transformation is important because it acts as a normalisation procedure that allows different samples to be compared. We eliminated all samples having more than 500 missing probe sets (which would result in a systematic bias towards higher ranks) or more than 500 probe sets with identical signal values (resulting in the random assignation of order ranks).

As RDAM can take advantage of duplicated measurements to evaluate the noise distribution and improve statistical power, we did not consider biological conditions represented by only one sample, and we selected exactly two replicates for all of the other biological conditions. We calculated the distance between any two samples (see Methods) and identified the two most similar replicates for each biological condition. We then inspected the scatter plots of the ranks of duplicates. Some duplicates were clearly of poor quality, e.g. with correlation coefficients smaller than 0.7, and the corresponding conditions were not used. Conversely, some duplicates were abnormally similar, e.g. with correlation coefficients greater than 0.98, and were discarded as well because they were technical replicates. Table 1 summarizes the effects of this first filtration step on the four chipset models used in this study: 18% of the experiments (24/134) and 32% of the biological conditions (302/945) were eliminated.

Following the first filtration step, we performed systematic comparisons between each pair of biological conditions. A visual inspection of the heat maps representing such comparison revealed that conditions with technical replicates or with poor quality duplicates stood out systematically from the rest by showing a higher or lower level of variation, respectively. The downloaded data were initially processed either with MAS4 or MAS5, the two main currently available versions of the Affymetrix analysis suite (see Methods). Comparison heat maps showed that the level of variations in MAS4/MAS5 comparisons was significantly higher than in MAS4/MAS4 or MAS5/MAS5. These observations led us to eliminate MAS4 samples from our systematic comparisons. Table 1 summarizes the effect of this final filtration step on the four chipset models used for this study: 38% of the experiments (51/134) and 47% of the biological conditions (447/945) were eliminated.

Another potential source of bias in the construction of CVMs is the presence of multiple experiments that contain similar biological conditions. In order to give the same weight to each biological condition and to eliminate redundant conditions, we constructed a distance matrix and defined as redundant those conditions that shared more than 80% identity. After this process, the number of biological conditions in chipset models HG-U95, HG-U133, MG-U74, and RG-U34 was 37, 84, 89, and 41, respectively (versus 71, 126, 205, and 96 at the completion of the quality filtering steps).

### Construction of CVM

Following the application of the quality filters described in the preceding section, we had multiple PxT matrices at our disposal for each chipset model, with P representing the number of probe sets and T the total number of comparisons. For example, the results of the analysis of the HG-U95 dataset can be synthesized under 12625 × 2485 matrices, since 71 biological conditions passed the quality filters (71 biological conditions allows for 71*70/2 = 2485 comparisons). Each matrix may contain one of the results produced by the RDAM analysis, such as the estimated total variation, the p-value, the false discovery rate (FDR), or the sensitivity. In these matrices, positive and negative values refer, respectively, to increased and decreased variations. From these raw values, we then constructed an oversimplified matrix in which variation was coded as either increased (I), decreased (D), or not changed (N). The decision rule necessary to make this transformation of numerical into symbolic values can be any expression that uses the statistical quantities assigned to each variation. In our case, we constructed two symbolic covariation matrices by using an FDR threshold value of either 10% or 1% (in the latter case, variations that had an FDR in the range -1 to -0.01; -0.01 to 0; 0 to 0.01; and 0.01 to 1 were labeled N, D, I and N, respectively). The number of symbolic matrices can be extended, if necessary, by considering, for example, conditions with a given property, e.g. a definite pathology or condition [32].

#### Calculation of CVM

The variation profile of a given probe set across all the comparisons is represented by an ordered series of symbols shown as a line in the symbolic matrix. If we extract two lines from the symbolic matrix, keeping them in register, and consider the different combinations of symbols in a particular comparison (column), we can encounter three different types of informative relationship between the two corresponding probe sets: the two probe sets can have the same type of variation (II or DD) and are thus said to be positively correlated (C); the probe sets can have opposite variations (ID or DI) and are said to be negatively correlated (A); or only one type of variation is ascertained (IN, NI, DN or ND) and they are said to be questionable (Q). The number of columns belonging to each of these classes (#C, #A and #Q) is counted and the corresponding percentages are referred to as Corr, Anti, and Quest (for example Corr = #C*100/T). These calculations, which allow the construction of three independent variables, are extended to all the possible pairs of probe sets, producing one matrix of positive correlation scores (the CORR matrix), one matrix of negative correlation scores (the ANTI matrix), and one QUEST matrix, with each matrix having the same PxP dimension.

**Figure 1:**
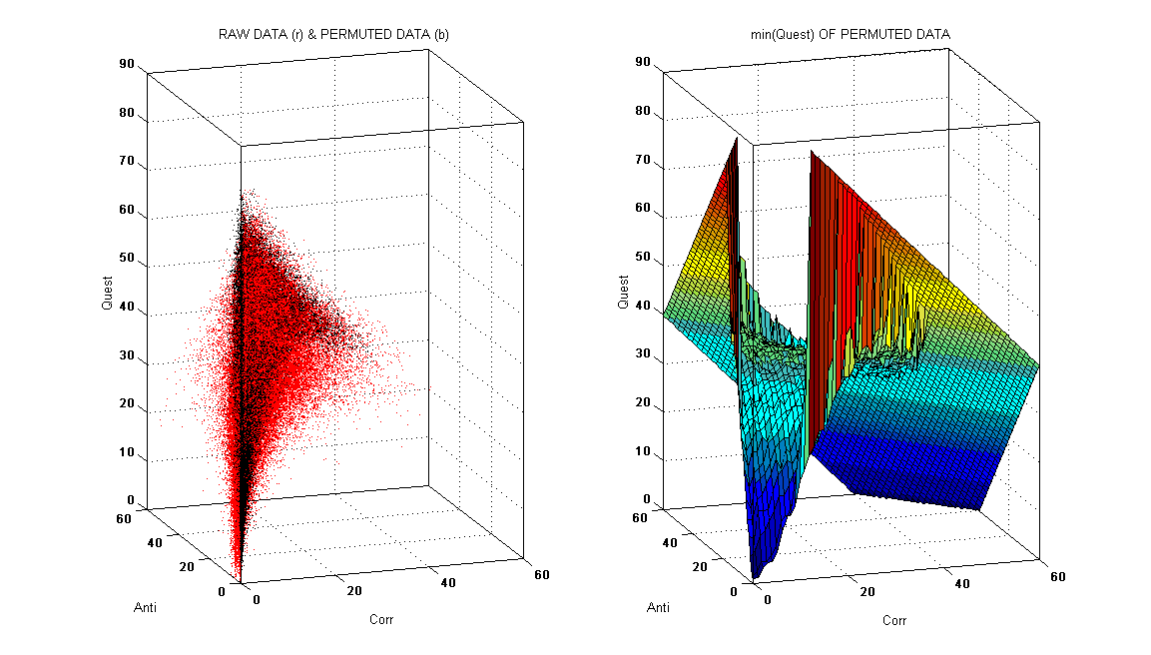
**Selection of statistically significant Corr and Anti values**. Left panel: Values corresponding to 10^6 ^pairs of probe sets of randomized and raw data are plotted as black and red points, respectively. Right panel: surface defined by the 5th minimum of Quest at a given (Anti, Corr) value and determined in 19 × 10^6 ^pairs of probe sets after randomization of comparisons.

Finally, we applied the same procedure to a randomized submatrix of size 1500xT in order to statistically eliminate non-significant values from the CORR and ANTI matrices. Because each line was randomized independently, the number of I, D, and N symbols in each line was conserved, but no significant positive or negative correlations could exist between any two lines (probe sets). The resulting CORR, ANTI and QUEST matrices could then be used to determine the noise and to extract the pairs of probe sets in which the Corr and Anti values were outside of the noise. Panel A of Figure 1 shows that for a given (Corr, Anti) coordinate the Quest value was consistently higher in the randomized comparisons than in the raw data. We therefore traced the surface defined by the 5th minimum of Quest for each (Corr, Anti) coordinate and set the Corr and Anti values of points located above this surface to zero. Then we re-calculated the Corr and Anti values that were outside of the noise, using informative relationships exclusively, in order to properly process probe sets that had significant variations in a small fraction of the comparisons. For example, if 5000 comparisons are considered, two probe sets that are positively and negatively correlated in 100 and 10 comparisons, respectively, and questionable in 90 comparisons will have their Corr values changed from (2% = 100*100/5000) to (50% = 100*100/(100+10+90)). The resulting CORR and ANTI matrices can be interpreted as networks: probe sets i and j, indexed by the ith line and the jth column, are interpreted as nodes, linked by two valued edges equal to CORR(i, j) and ANTI(i, j), respectively.

Table 2 shows that this procedure had a dramatic effect and eliminated a large fraction of the scores initially present in the CORR and ANTI matrices (from 75% for ALL10 networks to 97% for NR1 networks). The mean connectivity value was governed by three factors. First, the number of comparisons used had a positive effect; we observed that the mean ratio of ALL to NR mean connectivity values equalled 3.6 ± 2.3, a value comparable to the mean ratio of the number of comparisons between these two series of networks (4.2 ± 1.6). Secondly, the mean connectivity was dependent on the FDR and increased by a factor of 3 ± 1.44 when the FDR changed from 1% to 10%. Finally, the chipset model used also had a strong influence. Examining the mean connectivity normalized by the number of comparisons allowed us to classify the chips in the following order: Human Genome U95 Set, Rat Genome U34 Set, Human Genome U133 Set, and Mouse U74 Version2 (with 1.4, 0.3, 0.17 and 0.16 links per comparison, respectively).

**Table 2:** Statistical properties of networks

**chipset**	**network**	**#biol**	**#comp**	**#raw**	**#sel**	**%sel**	**mco**	**mc**	**mdc**	**ma**	**mda**
Human Genome	ALL1	71	2485	74017943	8400736	11.35	1331	36.21	22.38	13.31	13.86
U95 Set (12 6245 ps)	ALL10			78414854	16751383	21.36	2654	26.81	22.85	15.95	17.10
	NR1	37	666	70749360	2291090	3.24	363	42.10	20.86	29.07	21.03
	NR10			77866285	14260641	18.31	2259	29.63	23.43	20.01	20.91

Human Genome	ALL1	126	7875	182604317	7963686	4.36	715	35.71	23.98	9.10	8.81
U133 Set (22 283 ps)	ALL10			235733279	20713103	8.79	1859	29.37	22.47	11.90	12.27
	NR1	88	3828	160751542	3704369	2.30	332	47.68	30.04	9.12	8.25
	NR10			227849133	10331556	4.53	927	38.04	26.40	11.32	11.29

Murine Genome	ALL1	205	20910	75925711	11013118	14.51	1764	22.37	18.48	11.87	12.26
U74 Version 2 (12 488 ps)	ALL10			76566772	40596332	53.02	6502	20.63	21.28	18.71	20.37
	NR1	89	3916	74613508	2567219	3.44	411	38.18	28.10	11.91	12.12
	NR10			77231972	4633426	6,00	742	33.24	27.24	15.52	15.98

Rat Genome	ALL1	96	4560	25130304	2645806	10.53	601	36.69	26.01	16.07	16.64
U34 Set (8 799 ps)	ALL10			35636045	6508820	18.26	1479	29.99	23.92	16.69	17.58
	NR1	41	820	19937680	902388	4.53	205	55.05	46.33	23.35	23.26
	NR10			33301044	1801457	5.41	409	41.68	32.50	21.72	22.19

For each of the models listed, four networks were constructed based upon the combination of two independent factors: either all the biological conditions retained at the end of the two selection steps or only the non-redundant ones (labelled ALL and NR, respectively), and either 1% or 10% FDR (labelled 1 and 10, respectively). For each network, the table displays the number of biological conditions (#biol), the number of comparisons performed (#comp), the number of positive correlation scores in CORR (#raw), the number and percentage of statistically significant positive correlation scores (#sel, %s), the mean connectivity of a node (mco), and the mean and median positive (mc and mdc) or negative (ma and mda) correlation values linking a node to its neighbours. The values in the last five columns were computed on statistically significant correlation scores. The #raw, #sel and %sel values for the negative correlation scores in ANTI were similar to those of CORR and are therefore not indicated.

#### Performance of correlation scores

**Figure 2:**
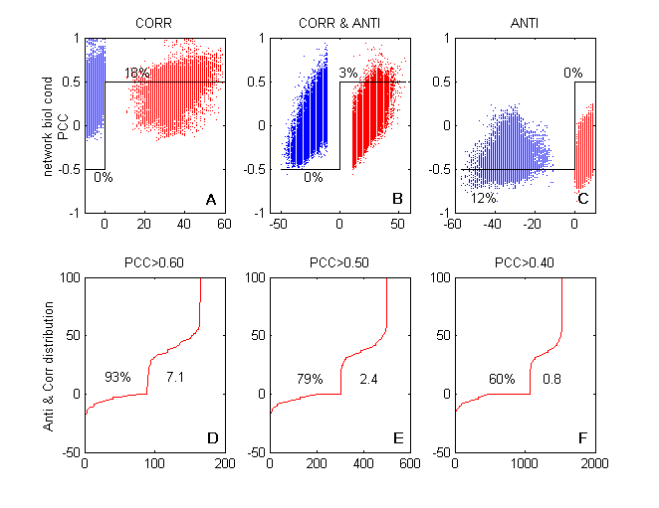
**Efficiency of detection by Pearson's correlation coefficient in three classes of probe set pairs**. **A, B, C**: We defined, in network HG-U133-NR10, three classes of probe set pairs that had positive and negative correlation scores of, respectively, > 10 and > -10 (mainly positively correlated and labelled CORR in **A**), > 10 and < -10 (positively and negatively correlated and labelled CORR & ANTI in **B**) and < 10 and < -10 (mainly negatively correlated and labelled ANTI in **C**). For each class we selected 30,000 random probe set pairs; the Figure indicates the percentage of the pairs that had a Pearson's correlation coefficient of greater than 0.5 or less than -0.5 (correlation coefficients were calculated on the ranks, but similar results were obtained with the log2 of signals). A probe set pair has its positive (red) and negative (blue) score plotted on the x-axis, and its Pearson's correlation coefficient plotted on the y-axis (a probe set pair is therefore represented by two points located on a line parallel to the x-axis). **D, E, F**: For the three subsets of probe set pairs whose Pearson's correlation coefficients were greater than 0.60 (**D**), 0.50 (**E**), or 0.40 (**F**), we have displayed the sorted values of the corresponding positive and negative scores ((a probe set pair is therefore represented by two points). The percentage of pairs that also existed in the network is indicated on the left of the curve, and the right side of the curve shows the ratio between the total number of pairs found to be positively correlated in the network and the number of pairs that would have been selected if the process of selection by Pearson's correlation coefficient, here applied to 30,000 probe set pairs, had been extended to the entire set of probe set pairs. Figures for negatively correlated probe sets were similar (not shown).

To examine the differences between our correlation scores and the widely used Pearson's correlation coefficients, we performed an evaluation using a cross validation-like approach. First, we defined three classes of probe set pairs, characterised by the different combinations of our positive and negative correlation scores. For each of these classes we calculated how many probe set pairs had the Pearson's correlation of their expression levels more than 0.50 or less than -0.50 (Figure 2, panels A to C). We noticed that the probe set pairs that had a marked imbalance between positive and negative scores were properly selected by the Pearson correlation coefficient method, albeit with a reduced efficiency. For example, 99% of the probe set pairs defined by us as mainly positively correlated in the HG-U133-NR10 network – i.e. with positive and negative scores respectively above 10 and -10 – also had positive Pearson's correlation coefficients. However, only 18% of these pairs had correlation coefficients that were greater than 0.50. The same is true for the mainly negatively correlated probe set pairs, but in this case the fraction of selected pairs was even smaller, with only 12% of them having a correlation coefficient of lower than -0.50. It is worth noting that the selection efficiency can be largely improved by defining more homogeneous classes. For example, using refined positively correlated probe set pair classes with negative scores of above -3 or equal to 0 instead of above -10 enables a selection efficiency of 25% and 60%, respectively. We can therefore infer that correlation coefficients applied directly to expression levels are very sensitive to the presence of small subsets of biological conditions in which the correlation is inverted relative to the main trend existing in all the other conditions. In contrast, our method is able to find about 25% of the probe sets pairs with positive and negative scores respectively greater than 10 and lower than -10, which remain undetectable when correlation coefficients are used (see panel B of Figure 2).

Second, we selected three subsets of probe set pairs which had Pearson's correlation coefficients of greater than 0.60, 0.50, or 0.40 (Figure 2, panels D to F), and estimated the fraction that belonged to the covariation network. In all cases, the retrieval rate using our method was high, reaching, for example, 80% when the probe sets were correlated at more than 0.50. In this particular case, however, we noticed that the network constructed using the correlation coefficient had 2.7 times fewer links than our network.

Based on these observations, we concluded that our method clearly outperforms methods based on the use of correlation coefficients in the selection of pairs of probe sets that are only partly correlated. Since the fraction of such probe sets is expected to increase in parallel with the number of biological conditions used to construct correlation matrices, this method is thus particularly well suited for the massive analysis of transcriptomic microarray results.

### CVM validation

To ensure that our method delivered reliable results, we applied several validation techniques. First, the information stored in a pair of positive and negative CVMs describes a network in which each pair of nodes is linked by at most two edges, corresponding respectively to a positive and a negative correlation, and taking their values in the ] 0,100] interval. In such a network or graph, the location of a particular node can be characterized by two neighbourhoods, i.e. the combination of all the nodes which are either positively or negatively correlated with this node. Comparing the neighbourhoods of probe sets targeting the same genes is a good approach for assessing whether CVMs constructed from different chipset models have high degrees of similarity. Secondly, we devised a technique to add a geometrical structure to the networks to visualise their internal structure. We observed that the correlation values did not obey the triangular inequality, i.e. f(anti(a, c), corr(a, c)) < = f(anti(a, b)corr(a, b))+f(anti(b, c), corr(b, c)), with corr(i, j) and anti(i, j) being the positive and negative correlations between nodes i and j, respectively, and f being a simple function calculating a value analogous to a distance by combining the positive and negative correlation values existing between i and j. Without such a property, a network remains an abstraction and cannot be represented in a 2D or 3D space in a realistic way. Once the nodes have been mapped to definite positions in space, the geometrical properties of the structure, such as the distance between a given node and its first neighbour, and structural properties such as its organization into clusters can be used to compare networks.

#### Topological network similarity assessed by measuring probe set neighbourhood similarity

A striking feature of the CVMs that we constructed is the variability of their mean connectivities in both intra- and inter-chip comparisons. For example, if we consider the NR10 networks, which we favour for most of our applications, the mean connectivity ranges from 409 (RG-U34) to 2259 (HG-U95), and the two human chipsets differ by a factor of 2.4, with 927 links for HG-U133 and 2259 links for HG-U95. Nevertheless, we expect that some kind of similarity exists across all the networks despite differences between the species and the biological conditions used.

To assess this similarity, we calculated, for each pair of probe sets targeting the same gene in two different chipset models, the probability that the observed overlap between their neighbourhoods occurred by chance. Letting C be the number of common probe sets between two chipset models, i be the rank of a probe set in a first network and j the rank of a probe set targeting the same gene in a second network, N1i and N2j be the number of neighbours for the ith and the jth probe sets in their respective networks (positive and negative correlations are treated independently), and I be the observed number of common probe sets between N1i and N2j, we calculated the probability of observing at least I common probe sets using the hypergeometric probability h(I, C, N1i, N2j) that the observed overlap is due to chance in the case of total independence between the two networks; we called this probability the neighbourhood similarity p-value.

**Figure 3:**
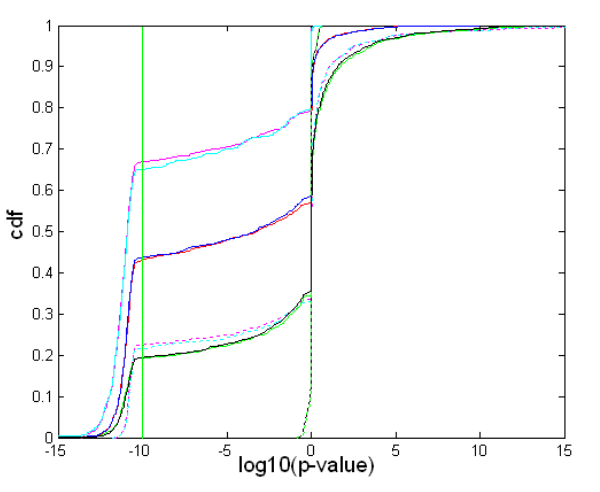
**Neighbourhood similarity for probe sets targeting a common gene**. The cumulative distribution frequency (cdf) of the logarithm of the p-values is plotted for the following categories of probe set pairs in network HG-U95-NR10: – unique pairs, i.e. genes targeted by exactly two probe sets (red: CORR, blue: ANTI). – multiple pairs, i.e. genes targeted by more than two probe sets. In this case, we considered either the best (magenta: CORR, cyan: ANTI) or worst p-values (magenta interrupted: CORR, cyan interrupted: ANTI). – random pairs, where the first probe set of unique pairs is matched with a probe set randomly selected from the second network (green: CORR, black: ANTI)- random network, where the first probe set of unique pairs is matched with a probe set taken from the second network after its neighbours have been randomized (green interrupted: CORR, black interrupted: ANTI). If the number of common neighbours is larger than expected, the log10(p-value) is calculated (left part of the curves, < 0), otherwise the opposite of the logarithm is calculated (right part of the curves, > = 0). A vertical line at log10(p-value) = -10 indicates the position of the inflection point used to tabulate the cdf values in Table 3. The presence of a strong inflection point at around -10 is an artefact of the algorithm, which is unable to calculate correctly very low p-values.

We were first interested in using this approach to compare probe set pairs targeting the same gene within a given chipset model. We assumed that examining such probe set pairs would allow us to eliminate any negative interference that might exist between two different chipset models due to the heterogeneity of the biological conditions used or to the observed inter-chip mean connectivity variability. The distribution obtained in these conditions should therefore give us a kind of standard that we could use to interpret the inter-chipset comparisons. Considering that more than two probe sets could target the same genes, we decided, in this case, to retain the two extreme p-values calculated for each of the possible probe set pairs combinations. Therefore, probe sets targeting the same genes were split into two categories: i) unique pairs (having only two probe sets) providing one series of p-values, and multiple pairs (having more than two probe sets) providing two series of p-values. As a control, we also constructed two randomized versions of the unique category: in the "random pairs" category, the second probe set was taken randomly, and in the "random network" category the second probe set had its neighbours randomized before calculating the p-value. The five distributions corresponding to these four categories are displayed in Figure 3.

**Figure 4:**
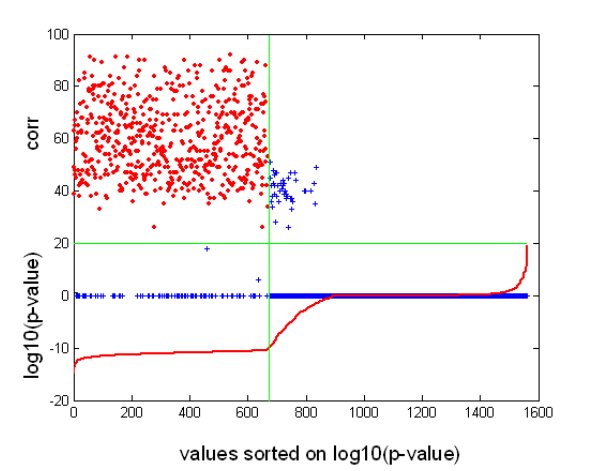
**Neighbourhood similarity p-value and positive correlation for pairs of probe sets targeting a common gene**. In the lower part of the figure is plotted (in red) the cumulative distribution frequency (cdf) of the logarithm of the neighbourhood similarity p-values for the unique pairs category in network HG-U95-NR10 (this curve corresponds to those of Figure 3, with an inversion of the x and y axes; the probe set pairs to the left of the green vertical line have a log10(p-value) < -10). In the upper part of the figure is shown the positive correlation score of the same probe set pairs. Red crosses indicate probe set pairs with a score > 20 and log10(p-value) < -10. Blue crosses designate probe set pairs with a score <= 20 or a log10(p-value) >= -10.

Plotting the correlation scores against the log10(p-value), as shown in Figure 4 for the unique pairs category, showed that most of the probe set pairs with a log10(p-value) of less than -10 were also related by a high positive correlation score; these two characteristics led us to consider them as truly targeting the same gene transcript. We hypothesized that the few pairs that had similar neighbourhoods but with correlation scores equal to zero were composed of probe sets targeting two alternatively spliced transcripts of the same gene, with different regulation (preliminary results not shown). We decided to use this log10(p-value) limit of -10 to determine, in Tables 3 and 4, the fraction of probe set pairs that could be considered consistent on the basis of their high neighbourhood similarities.

**Table 3:** Fraction of consistent probe set pairs targeting the same gene.

		**random pairs**		**unique pairs**		**multiple pairs**		**< -10**
chipset model	network	#pairs	%sel	#pair	%sel	#pairs	%sel	%com

HG-U95	NR1	1537	11	1519	32	625	48	66
HG-U95	NR10	1563	19	1561	43	633	67	50
HG-U133	NR1	2721	8	2653	33	2023	56	47
HG-U133	NR10	2821	20	2814	44	2061	72	37
MG-U74v2	NR1	1442	12	1438	41	336	62	62
MG-U74v2	NR10	1460	18	1460	51	337	81	46
RG-U34	NR1	1110	10	1075	39	507	57	74
RG-U34	NR10	1144	19	1141	49	521	71	59

For each CORR network, the table indicates the number of probe set pairs considered (#pairs) and the percentage of probe set couples (%sel) with a log10(neighbourhood similarity p-value) of less than -10 in three categories of probe set pairs: labelled random pairs, unique pairs, and multiple pairs (see Figure 3 legend). For the multiple pairs category, the probe set pair having the best p-value is retained. The final column (%com) presents the mean percentage of links that are common between two sub-networks constructed by considering probe sets with more than 100 links and by picking the first or the second probe set from all the probe set pairs with a log10(p-value) < -10. ANTI network values are very similar.

**Table 4:** Fraction of consistent probe set pairs targeting the same gene in two networks.

			**CORR**			**ANTI**	
chipset model 1	chipset model 2	network	#pairs	%sel	%com	#pairs	%sel

HG-U95	HG-U133	NR1	4600	64	40	4143	42
HG-U95	HG-U133	NR10	4769	67	34	4605	52
HG-U95	MG-U74v2	NR1	2061	53	45	1914	27
HG-U95	MG-U74v2	NR10	2093	50	42	2057	32
HG-U95	RG-U34	NR1	1603	51	65	1433	18
HG-U95	RG-U34	NR10	1653	48	60	1602	21
HG-U133	MG-U74v2	NR1	3285	65	45	3002	38
HG-U133	MG-U74v2	NR10	3332	65	37	3268	47
HG-U133	RG-U34	NR1	2230	58	65	1886	25
HG-U133	RG-U34	NR10	2316	58	54	2214	33
MG-U74v2	RG-U34	NR1	1503	63	59	1345	34
MG-U74v2	RG-U34	NR10	1554	59	51	1511	41

The fraction is calculated with the best value of the multiple pairs category. For each comparison between two networks, the table indicates the chipset model of the first and second network (chipset model 1 and 2), the name of the networks (network), the number of probe set pairs considered (#pairs), and the percentage of probe set pairs (%sel) with a log10(neighbourhood similarity p-value) of less than -10 in networks constructed either on positive (CORR) or negative CVMs (ANTI). The column (%com) indicates the percentage of links shared between two sub-networks constructed by examining probe sets with more than 100 links and picking the first or second probe set from all the probe set pairs having a log10(p-value) < -10.

It is worth noting that the random pairs have a notable fraction of consistent pairs (10.2 ± 1.7 in NR1 networks and 19 ± 0.8 in NR10 networks, compared to 36.2 ± 4.4 and 46.8 ± 3.9 for the unique pairs). At least two explanations could account for the high level of consistency observed between the randomly selected probe sets. First, the networks under consideration are scale-free (results not shown) and contain several hubs connected to a large proportion of the other nodes. As a consequence, two probe sets selected at random have a high probability of counting most of these hubs as neighbours. Second, the high mean connectivity (Table 2) means that the network can be subdivided into a large number of overlapping subsets that have a high degree of inter-connectivity – an analysis technique known as biclustering- and the probability that two random probe sets will be found in one of these subsets is therefore not negligible. Finally, it can be seen that the heterogeneity within multiple pairs is very high, since the worst p-value curves are superimposable on those of the random pairs (Figure 3), and the fraction of consistent pairs calculated from the best p-values is 1.5 times the fraction of unique pairs (55.8 ± 5.8 in NR1 networks and 72.8 ± 5.9 in NR10 networks, compared to 36.2 ± 4.4 and 46.8 ± 3.9 for the unique pairs). In view of this analysis, we conclude that the fraction of consistent pairs calculated from the best p-values of multiple pairs is the right statistical tool to use to estimate the neighbourhood similarity between two networks, because it increases the fraction of pairs suitable for this type of comparison. We expect that the fraction of consistent pairs that can be obtained when testing for the similarity of two networks is around 55% and 70% for the NR1 and NR10 networks, respectively.

The presence of numerous consistent pairs – i.e. with a log10(p-value) < -10 – allowed us to construct two sub-networks with pair-wise correspondence between all of their nodes by randomly assigning each member of a pair to one of the sub-networks. Counting the fraction of links that is conserved between these two sub-networks is another way of measuring the overall reproducibility of the methods. We found that the mean reproducibility ranged from 42% to 88%, depending on the connectivity of the nodes considered (respectively > 0 and > 200). The final columns of Table 3 and Table 4 show one example of such a measure, for nodes having a connectivity of greater than 100 (see additional file 3).

We used the same approach to compare matched networks constructed in two different chipset models. The results obtained were similar to those displayed in Figures 3 and 4 for intra-chip comparisons (result not shown); Table 4 presents the numerical values obtained measuring the neighbourhood similarity. The first conclusion we were able to reach from this analysis was that the similarity of the networks across different chipset models and/or different species remained very high: the fraction of consistent probe set pairs was 59.0 ± 6.0 and 57.8 ± 7.7 for the NR1 and NR10 CORR networks, respectively. It is worth noting that the mean best value obtained by matching different chipset models (59 ± 6) in the NR1 CORR networks was directly comparable to the best value obtained within individual networks (55.8 ± 5.8). This means that the 1% FDR selection truly succeeded in finding positive correlations, which were conserved across the three species studied independently of the set of biological conditions used. Indeed, this level of conservation drops when a 10% FDR selection is applied (from an expected value of 70% to an observed value of 58%); we may suppose that this is a direct consequence of the disparity among the biological conditions used. Considering that this effect was of the same magnitude in the comparison between the two human networks and in inter-species comparisons, we conclude that differences in regulation between species cannot be easily detected if the networks are not normalized with respect to their biological conditions.

Taken together, all of these observations indicated that some kind of invariant topological organization underlies the structure of all of the networks that we constructed. However, as we only considered the first neighbours of each probe set, i.e. all the probe sets that were correlated with it, we cannot conclude that this shared organization exists at every scale. It seems sensible, however, to think that as long as small scales are considered our networks are congruent. Finally, we observed that there was a clear difference, which had not been observed in our previous study restricted to single networks, between the CORR and ANTI fraction values, with the CORR values being greater by 26 ± 6 points (NR1) and 20 ± 5 points (NR10). We can therefore speculate that negative correlations are far more sensitive to the nature of the biological conditions used to construct the network than are positive correlations, and that the former have greater weight in the shaping of networks.

#### Geometrical network similarity assessed by measuring probe set neighbourhood distance and degree similarity

**Figure 5:**
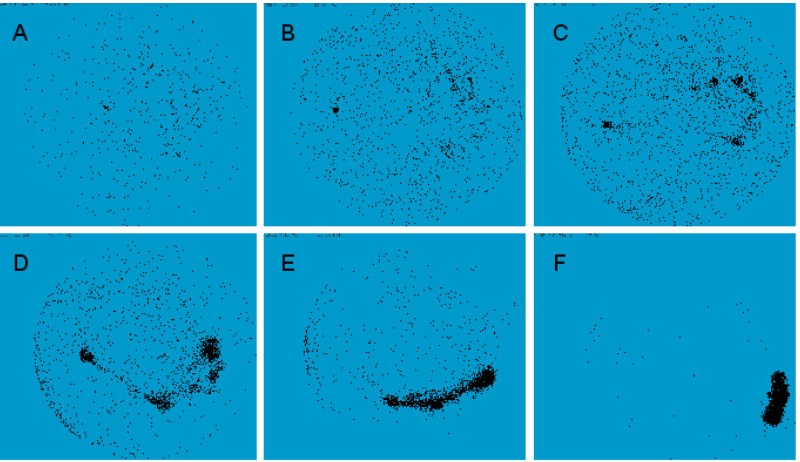
**Mapping of probe set position in 3D space with Keiko**. Each panel shows the configuration of nodes after attractive forces greater than 60 (**A**), 50 (**B**), 40 (**C**), 30 (**D**), 20 (**E**), or 1 (**F**) have been activated and an equilibrium obtained (network HG-U95-NR1). For repulsive forces, the threshold used to activate the forces is equal to the attractive force minus 10. Each node is allowed to move in the direction resulting from the sum of the attractive and repulsive forces, and the whole network reaches a new configuration characterized by its potential energy. When the difference in energy between two successive configurations is inferior to a given threshold, new forces with smaller values are activated. Nodes are not allowed to collapse, and repulsive forces are automatically added when the distance between two nodes is below a given limit.

The geometrical representation of a network in 2D or 3D space is another important point to be considered. To map each node to a particular set of coordinates, we devised an algorithm called Keiko which uses a physical paradigm. For a given pair of probe sets, we calculate the difference between the positive and negative correlation scores and interpret the resulting positive or negative values as an attractive or repulsive physical force, respectively. We then place the nodes randomly on the periphery of a 3D universe and let the attractive and repulsive forces act incrementally by repeatedly activating forces restricted to a given range, starting with the highest absolute values and finishing with the lowest (see legend to Figure 5). As this aggregation process advances, the discrete clusters which are present at the beginning get progressively closer, ultimately forming what we call a probe set map (not shown). We show in Figure 5 six successive steps of this aggregation process, showing that the discrete foci which appeared at the very beginning (panel A) got progressively closer (panels C and D) and finally formed a clump at the end of the process (panels E and F). In each network, numerous probe sets stayed outside of the final probe set map (representing 34%, 41%, 24%, and 47% of the probe sets for the HG-U94, HG-U133, MG_U74v2, and RG-U34 chipset models, respectively).

**Figure 6:**
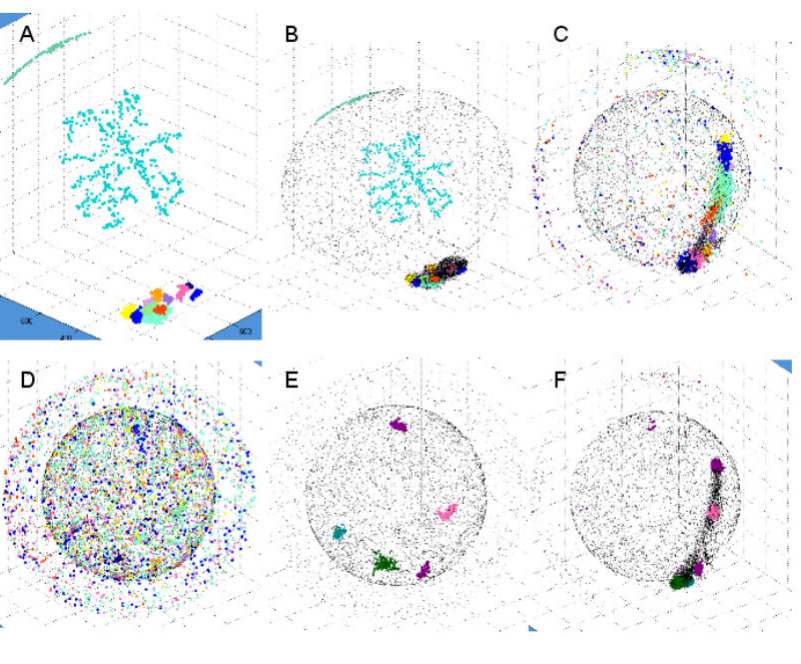
**Clustering of probe sets with Gene DIVER**. Eleven clusters found by Gene DIVER on HG-U133-NR1 in step 1, the final step of the mapping process by Keiko (**A**, **B**, corresponding to without and with all other non-clustered probe sets, respectively) are represented at step 30 (**C**) and step 60 (**D**). A and B show one cluster (in cyan) that is not significant because it is composed of probe sets that were not activated and that stayed randomly positioned at the periphery of the 3D universe. Five clusters found by Gene DIVER on HG-U133-NR1 at step 60 of the mapping process by Keiko (**E**) are represented at step 30 (**F**).

To check the continuity of the structures that appeared in the initial steps, we used the software Gene DIVER [33] to delimit dense regions buried in a sea of unorganized points and observed how these clusters of points evolved throughout the process. As shown in Figure 6, clusters that appeared in the first steps were maintained in the final configuration; we thus concluded that our method allows probe sets to follow stable trajectories.

**Figure 7:**
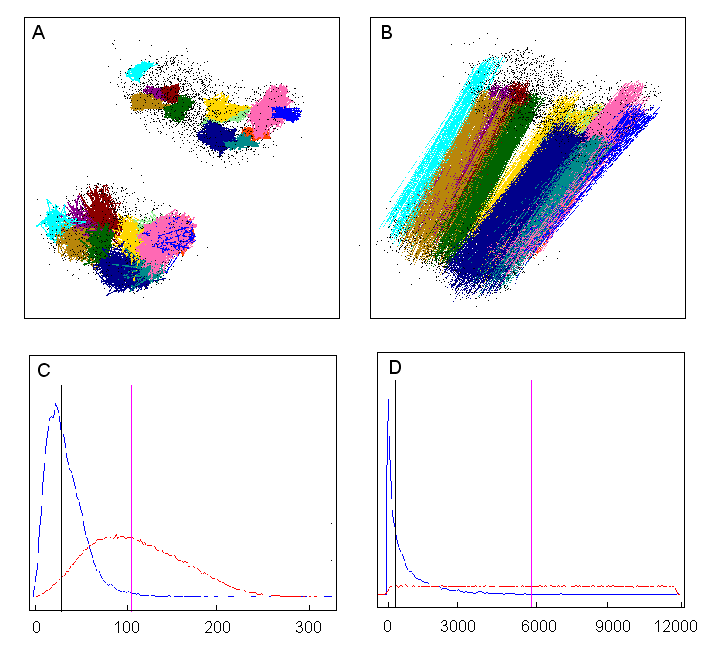
**Reproducibility of Keiko 3D mapping**. Two Keiko probe set maps constructed on HG-U133-NR1 are compared (replicates a and c of Table 5). **A **– Twelve clusters found by Gene DIVER in replicate c are displayed in the upper right corner. The corresponding points on replicate a are displayed in the lower left corner. All the points belonging to a given cluster in replicate c are linked by a coloured line; the same is true for the corresponding points in replicate a. **B **– Two points corresponding to a pair of probe sets are linked by a line coloured according to the cluster they belong to. **C **– In blue is plotted the probability density of the distance in replicate a between one point and the closest point (i.e. its rank is equal to 1) in replicate c. In red is plotted the probability density of the mean distance in a series of random permutations of replicate a. The black vertical line shows the median value of the first distribution. The magenta vertical line indicates the mean of the 100 median values calculated on permuted maps. The ratio between these two values was used to calculate the distance score, which represents the "distance similarity" between the two maps. **D **– In blue is plotted the probability density of the ranks in replicate a between one point and the closest point in replicate c. In red is plotted the probability density of the mean ranks in a series of random permutations of replicate a. The ratio between the two values marked by the two vertical lines was used to calculate the ranking score, which represents the "rank similarity" between the two maps.

Another issue that we addressed was the influence of the random starting positions of points on the reproducibility of the process. We constructed several Keiko maps from one network and compared these maps to one another. To compare any two maps and assess the reproducibility of the network geometry, we first searched, for each node, the closest node in a map used as a reference, which was given a rank equal to one; we then measured in another test map the distance and the rank which separated the two corresponding nodes. A distance score was calculated by determining the ratios of the median distances before randomization of the test map to the median distances after randomization (see panel C of Figure 7). Similarly, a ranking score was calculated by determining the ratios of the median neighbourhood degrees (see panel D of Figure 7). The upper parts of Table 5 and Figure 7 show that the process is largely reproducible, although the random positions of the points at the start of the process introduce some local fluctuation.

**Table 5:** Reproducibility and similarity of the geometrical structures.

	**distance score**		**ranking score**	
comparison	mean	std	mean	Std

HG-U133 rep a vs rep b	4	0.03	20.62	0.22
HG-U133 rep a vs rep c	4.22	0.03	27	0.3
HG-U133 rep a vs rep d	4.09	0.03	24.43	0.26
HG-U95 vs HG-U133	2.36	0.06	4.12	0.15
HG-U95 vs MG-U74v2	1.93	0.05	3.56	0.15
HG-U95 vs RG-U34	2.6	0.17	3	0.23
HG-U133 vs MG-U74v2	1.9	0.04	3.61	0.14
HG-U133 vs RG-U34	3.1	0.13	3.63	0.2
MG-U74v2 vs RG-U34	3.36	0.14	3.5	0.2

The three top lines show the geometrical and topological scores calculated between replicates of the HG-U133-NR1 Keiko map, giving an indication of the reproducibility of the mapping process. The lower part of the table shows the scores calculated between maps constructed on different chipset models. The scores were calculated as indicated in the legend to Figure 7.

**Figure 8:**
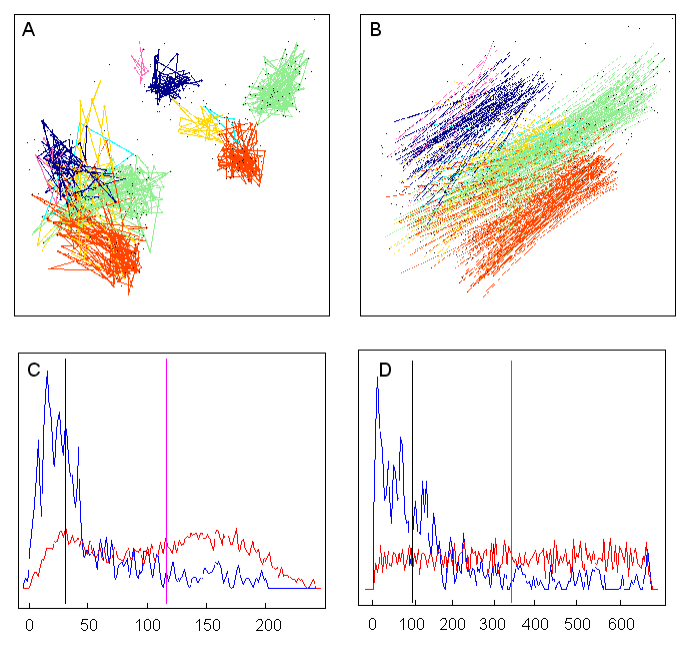
**Geometrical and topological network similarity**. Two Keiko maps are compared, one constructed on MG-U74v2-NR1 and another on RG-U34-NR1. Only unique and multiple probe set pairs are taken into account in the display and statistics. **A **– Six clusters found by Gene DIVER in the RG-U34-NR1 map are displayed in the upper right corner. The corresponding points on the MG-U74v2-NR1 map are displayed in the lower left corner. All the points belonging to a given cluster in the RG-U34-NR1 map are linked by a coloured line, and the same is true for the corresponding points in the MG-U74v2-NR1 map. **B **– Two points that correspond to a pair of probe sets are linked by a line coloured according to the cluster they belong to. **C **– In blue is plotted the probability density of the distance in the RG-U34-NR1 map between one point and the closest point in the MG-U74v2-NR1 map. In red is plotted the probability density of the mean distance in a series of random permutations of RG-U34-NR1. The black vertical line indicates the median value of the first distribution. The magenta vertical line marks the mean of the 100 median values calculated on permuted maps. The ratio between these two values is used to score the "distance similarity" between the two maps. **D **– In blue is plotted the probability density of the ranks in the RG-U34-NR1 map between one point and the closest point in the MG-U74v2-NR1 map. In red is plotted the probability density of the mean rank in a series of random permutations of RG-U34-NR1. The ratio between the two values marked by the two vertical lines is used to calculate a ranking score that scores the "rank similarity" between the two maps.

The same technique, applied to the comparison of maps constructed from different chipset models, allowed us to confirm that the high level of similarity that we had observed on networks, at least at a small scale (Table 4), was still present when these networks were mapped to a definite 3D configuration with Keiko (Figure 8 and Table 5). Indeed, Figure 8-A shows that clusters of points located in any given region of the map in a particular network remained grouped when mapped in another network. Visual inspection of Figure 8-B shows that the lines linking a given probe set in two different networks are not strictly parallel. Taking into account that the distance or rank between a given probe set and the closest different probe set does not change dramatically when measured in a matched network (Table 5), we can conclude that the topological similarity detected in the previous section – most of the first neighbours of a probe set in a given network are also first neighbours of the same probe set in a matched network – is reflected by a geometrical similarity – the closest probe set to a given probe set in one network stays close to the same probe set in a matched network – when our mapping procedure is applied.

### Structural and functional organisation of the networks

The above analysis showed that our methods succeeded in constructing networks whose geometrical and topological properties were well conserved over a short range. In this section, we show how larger conserved functional regions can be defined and how a synthetic view of the large scale structure of a network can allow conclusions to be drawn about the general organization of the network.

#### Large scale structural organisation of the networks

The clusters found by Gene DIVER in the Keiko probe set maps could have been used to study the large scale structural organisation of the networks, as it was possible to identify clusters sharing the same genes across the four networks (results not shown). However, as local density is not an invariant characteristic of Keiko maps (Figure 7), we preferred to concentrate instead on finding a more robust definition of density that was independent of any geometrical representations, using the clustering coefficient to this effect. In this case the clusters are formed by probe sets which are linked together by a high number of edges. The Markov clustering algorithm [34,35] is designed to find this type of cluster by simulating a random walk inside the network and interpreting the strength of a link – in our case the difference between the positive and negative correlations between two probe sets, set to zero if negative or below a given limit – as the probability of following that link. We observed that clustering networks using limits of 0, 10, or 20 gave comparable results, and that clustering with too high of a limit, e.g. 50 or 60, resulted in too few probe sets being located in clusters of reasonable size. We therefore used limits of 0, 30, and 40 and compared, for each limit, the clusters obtained in the four networks constructed with a 1% FDR. We determined a set of six common regions, with each region possibly resulting from the merging of several clusters in a particular network (see Methods), combining 71%, 60%, 67%, and 68% of the probe sets present in the probe set maps of the HG-U94, HG-U133, MG_U74v2, and RG-U34 NR1 networks, respectively. All of the probe sets belonging to clusters not used in the construction of the regions and all of the non-clustered probe sets belonging to the probe set map were assigned to regions 7 and 8, respectively, for the sake of convenience (these subsets are not proper regions since they are not constructed on the basis of common content across the four networks).

**Figure 9:**
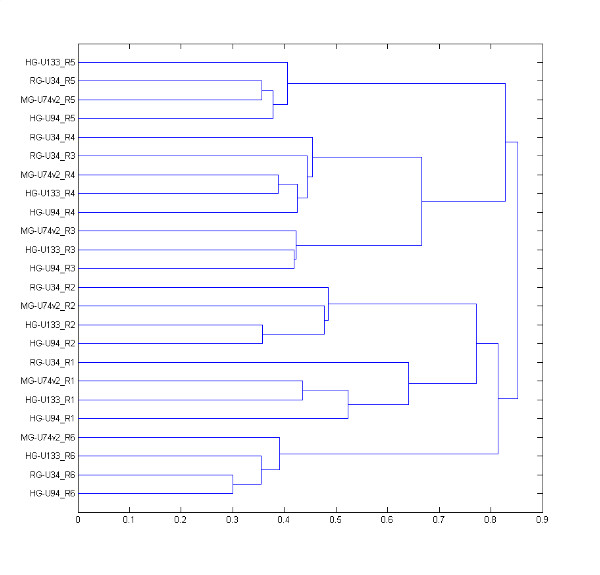
**Dendrogram of the six common regions found in the four NR1 networks**. The regions are named as follows: at the beginning the name of the chipset model is indicated, followed by the region number. The distance used to measure the closeness of two regions was taken as equal to 1 – Inter(1,2)/min(1,2), where Inter(1,2) is the number of probe sets common to the two regions and min(1,2) is the minimum of probe sets in the two regions. For example, MG-U74v2 and RG-U34 have 745 and 1041 probe sets, respectively, in region 5, of which 410 and 785 are present in the alternate chipset model. As MG-U74v2_R5 and RG-U34_R5 have 264 probe sets in common, we calculated a distance equal to 1 – 264/410 = 0.35, which reflects the 70% of the probe sets they have in common.

The dendrogram of Figure 9 shows that all of the regions, except for region 3 of RG-U34, are perfectly grouped with respect to their ranks, and that each group of regions is well separated from the others, meaning that we succeeded in delineating six different regions that are well conserved in the four networks. For the six regions, we measured the mean percentages of conserved probe sets to be 39% ± 12, 48% ± 11, 29% ± 23, 53% ± 5, 57% ± 6, and 60% ± 6, respectively.

The probe sets located in each region are highly connected, with a mean connectivity of 0.44 ± 0.17 and 0.42 ± 0.17 for the positive and negative correlations (the connectivity of a region is equal to the number of edges it contains divided by the total number of edges it could contain, and the mean is calculated over all six regions). Another characteristic of the regions is the large fraction of edges emanating from the probe sets located in a given region that target probe sets located in the same region (0.40 ± 0.13 of links stay inside the regions). The equality of the positive and negative connectivity does not mean that the two types of correlation have the same importance inside the regions. In fact, we found that positive correlations were predominant inside the regions, having a mean value of 40 ± 6, which was much greater than the negative correlations, which were measured at 7 ± 3. In comparison, calculating the same values between a given region and the other five regions showed that positive and negative correlations had the same weight: the correlation values were equal to 22 ± 5 and 23 ± 6, respectively, and the connectivity was equal to 8 ± 4 in both cases (see additional file 4 for complete results). From these averaged properties, we can characterize the network as a structure comprised of six loosely connected regions that contains highly connected probe sets with high positive correlation and low negative correlation.

**Figure 10:**
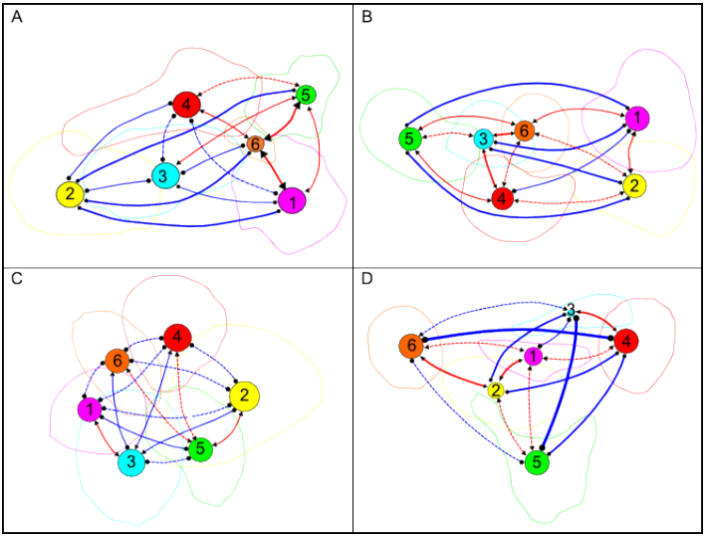
**Mapping of regions found with the Markov clustering algorithm onto Keiko probe set maps**. A: HG-U95, B: HG-U133, C: MG-U74, D: RG-U34. Each of the six regions corresponds to a circle whose size is proportional to the number of probe sets it contains. Regions one to six are colour-coded as magenta (immunity), yellow (nucleus), cyan (adhesion), red (energetic metabolism), green (nervous system), and orange (metabolism), respectively. The extent to which the probe sets belong to a given region is indicated by a loop of the same colour. Red and blue lines indicate the inter-cluster correlation trends. Negative correlation trends – a condition in which the mean of the negative correlation values is greater than the mean of the positive correlation values – are rendered by blue lines, and positive correlation trends by red lines (see section V of Methods). The dashed lines correspond to trend >= 1 and < 2, and the three increasing widths of the continuous lines correspond to increasing strength values (respectively to range 2–3, range 3–5, and >= 5).

By mapping all the probe sets belonging to a given region onto the probe set maps constructed with Keiko, we found that the corresponding points were also localized to a defined region made up of a dense subset of points localized in a small part of the whole space, with very few intersections with the other regions. This means that the application of two very different algorithms produced two congruent outcomes: either a clustering of probe sets by Markov clustering, or a disposition of the probe sets in a 3D space by the Keiko algorithm. The combination of the two types of information – regional membership and position – allowed us to obtain a synthetic view of and have a more precise perspective on the structural organisation of the networks coded inside the CVM. We calculated for each pair of regions an inter-region correlation trend (see section V of Materials). Each region was then displayed both as a circle positioned at the barycentre of its probe sets and as a closed line delimiting its maximal extension. The positive and negative correlation trends are represented by red and blue lines, respectively, whose widths reflect the trend strength. Figure 10 displays the four maps obtained with this procedure.

We observed, first, that the probe set map architecture is shaped by both attractive forces, which are responsible for the formation of dense regions of probe sets and for the proximity of some regions, and repulsive forces, which are responsible for the separation of some regions. For example, the HG-U95 and HG-U133 networks, which have strong parallel negative trends, are outstretched (Figure 10-A and 10-B), while RG-U34 is constrained into a triangular shape by strong orthogonal negative trends (Figure 10-C). Conversely, as most of the inter-region trends in MG-U74v2 are slightly negative, it is not surprising that the corresponding structure is symmetric and round (Figure 10-D). Another interesting observation is that positive correlation trends are not transitive over long distances: if regions A and B are close and positively correlated, and so are regions B and C, then regions A and C have a positive correlation trend as well. But if we add a more distant region D that is positively correlated with, e.g. C, then regions A and D may have a negative correlation trend, as exemplified by regions 1, 4, 6, and 5 of Figure 10-A. In other words, a chain of positively correlated regions is generally terminated by negatively correlated regions.

Finally, we note that it is impossible to obtain a consensus description of the network structure: six well-defined regions, each composed of highly correlated probe sets, are indeed present in all four networks, but the relationships between these regions are not constant over the four networks, and some regions that are mainly negatively correlated in a given network are positively correlated in another network, e.g. regions 1 and 2 in the HG-U95 and HG-U133 networks. Given the high similarity between the networks demonstrated above, we conclude that their organisation into six regions is a strong and fundamental characteristic, whereas their observed relationships are largely dependent on the particular subset of biological conditions used to construct them.

#### Small scale structural organisation of the networks

**Figure 11:**
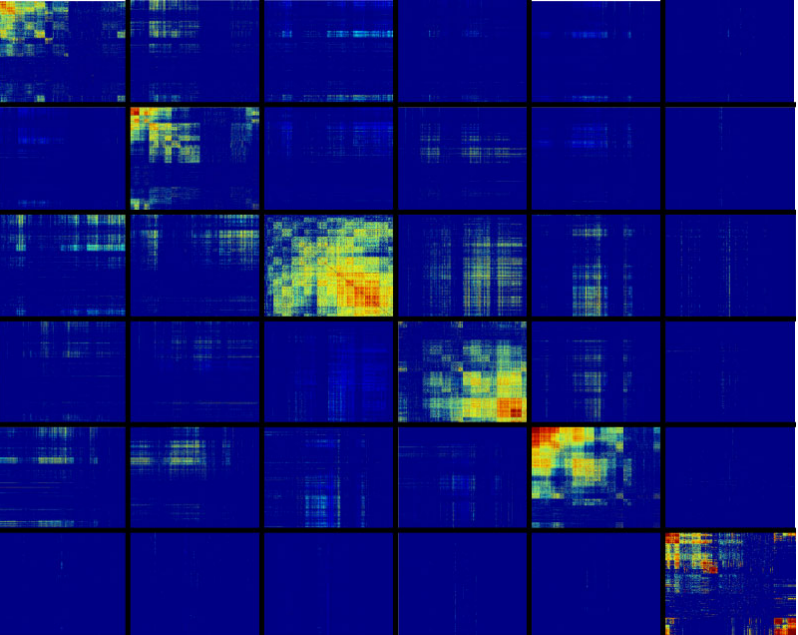
**Structure of the HG-U133 NR1 positive and negative CVMs**. We clusterized positive correlation measures independently in each region using annealing clustering. The six regions, ordered on a diagonal from top left to bottom right, display the positive correlation values between the probe sets belonging to each region. Shown above and below this diagonal are the positive and negative correlation values between probe sets belonging to different regions.

The structure of the core network, i.e. composed of six regions, becomes more apparent when the CVMs are displayed after having been reordered by clustering positive correlation measures independently in each region using annealing clustering [36], as shown in Figure 11 for the HG-U133-NR1 CVM (see additional file 5 for a representation of all the CVMs). In accordance with what is shown in Figure 10 and with the conclusions of the preceding section, this representation shows that the six regions are loosely connected. In addition, the clustering makes it obvious that the probe sets are organized within each region into overlapping groups of tightly correlated probe sets – a characteristic of functional modules- and that the interactions between the modules within a given region are strong. However, we were unable to find a simple way of identifying modules that were common to the four networks; better analytic techniques and/or normalization of the biological conditions could potentially show that the different networks are similar at this level of organization. It can also be seen in this particular example that in regions 2, 3, and 4 most of the probe sets are present within modules, whereas in the other three regions about one-third of the probe sets are sparsely correlated. It might not be coincidental that these sparsely correlated probe sets are never implicated in positive correlations between the regions, and that only probe sets within modules can have both positive and negative correlations between the regions.

#### Functional organisation of the networks

We used the GENERATOR tool (Pehkonen et al., 2005) to calculate p-values for all the GO terms associated with the genes present in the different regions, and asked whether the regions were homogeneous with respect to their Gene Ontology annotations. By considering the five GO terms with the most significant mean p-values, we were able to determine, without ambiguity, the main characteristics of each bona fide region within the three branches of the GO nomenclature. To give a short description for each region, we selected the five most representative terms from the three GO branches (i.e. biological process, molecular function, and cell component; see the "Fist5GO" sheet in the additional file 6)) and constructed the following descriptors, using one representative term for each GO branch, corresponding respectively to regions one to six: immune system/signal transduction/lysosome (1), nucleic acid metabolic process/nucleic acid binding/nucleus (2), cell adhesion/extracellular matrix structural constituent/extracellular matrix (3), energetic metabolism/NADH dehydrogenase activity/mitochondrion (4), nervous system/ion channel activity/neuron (5) and metabolism/oxidoreductase activity/endoplasmic reticulum (6). The six regions are therefore related to the immune system, nuclear processes, cellular adhesion, energetic metabolism, the nervous system, and metabolism, respectively.

Several observations allowed us to conclude that these six regions form a well-established structure that is present in all four networks when studied at a functional level. First, most of the terms with a p-value of <= 0.01 (OLog >= 2) were directly or indirectly related to the main characteristic of their region. Second, the characteristic terms had extremely low p-values and were present in a high proportion of the genes. Third, we calculated two scores for GO terms having p-values equal or inferior to 0.001 (OLog >= 3) for each region and each network, in order to assess the degree of homogeneity in each region; we found that the similarity score, which is the percentage of GO terms found in at least three networks, and the purity score, which is the percentage of GO terms specific to the region under consideration, were both high, with means of 97% and 73%, respectively (see the "GOScores" sheet in the additional file 6 for complete results). We also found, however, that three regions of the rat network were not really pure and were enriched in genes normally assigned to other regions in the three other networks: region 1 was enriched in GO terms associated with region 3 (more specifically related to collagen metabolism and cartilage development), and regions 2 and 3 contained many terms linked to region 1 (immunity).

Another function of GENERATOR allows the clustering of genes to groups that have GO terms in common. We constructed another score that measures the number of clusters containing a given GO term. This information allowed us to detect groups of genes with very specific functions (see the "GOClustering" sheet in the additional file 6). For example, the GO term "DNA unwinding" was very specific and was found only eight times in the list of GO terms corresponding to the human chipsets. In HG-U94 and MG-U74, this term was found in two clusters: one cluster of 138 and 114 genes, respectively, mainly related to transcription (clusters 17 and 14), and one cluster containing 60 and 53 genes, respectively, linked to the regulation of chromatin and DNA packaging (clusters 2 and 10). In HG-U133 and RG-U34, all the "DNA unwinding" terms were found in one cluster per dataset, containing 119 genes (cluster 7) and 14 genes (cluster 4), respectively, related to chromosome organization. This score also confirmed the difference between regions 4 and 6. By listing the terms with the highest mean scores calculated for the four networks, we found that in region 4 all the terms were related to energy production (e.g. glycolysis, fatty acid beta-oxidation, alcohol catabolic processes, respiratory chain complex I, cellular respiration), while in region 6 most of the terms were connected to specialized catabolic or anabolic functions (amino acid catabolic processes, nitrogen compound catabolic processes, tetrapyrrole binding, fibrinogen complex, protease inhibitor activity, lipid transporter activity, peroxisome).

## Discussion

Our study highlights the importance of doing quality controls and underlines the effects of various parameters on the structure and quality of the constructed networks. A typical example is the dramatic reduction in mean connectivity, which was reduced by a factor of three when the redundancy of biological conditions was taken in account (cf table 2). Another example is the presence of artefactual variations, which were observed in comparisons between data analyzed by two different versions of the Affymetrix software. The significant impact of the analysis method used raises particular concern since there is no required field for indicating the analysis method used when data are deposited on the GEO repository site. We are confident that all of the data we ultimately used were analysed by MAS5 because they were all uploaded by the end of 2005, a time when alternative analysis methods were not frequently used. In a recent paper, Lim WK et al. [37] estimated the impact of normalization procedures on the correlation structure. Testing the hypothesis that highly co-expressed gene pairs are more likely to share common GO terms and to predict protein-protein interactions than are uncorrelated genes, they showed that the MAS5 algorithm outperformed the RMA [38] and Li-Wong methods [39], and also that the GRCMA methods [40] were of little use because they introduced many correlation artefacts. In view of this evidence, it seems advisable to work directly on raw data (Affymetrix CEL files) and to normalise the probe set signal computation by applying the same adapted algorithm. The Celsius project, which is designed to be a repository of raw Affymetrix data and of various analytic methods, makes this approach perfectly realistic [41]. Independent of the algorithm used to calculate the pair-wise correlations, normalisation is certainly one of the most important steps for attaining a high degree of reproducibility when networks constructed in different species are compared. Accordingly, we think that our results could be greatly improved by normalising the network against the targeted transcripts. The statistical study of probe set neighbourhoods (Figures 3, 4 and Tables 3, 4) shows that two probe sets assigned to the same genes can have divergent neighbourhoods. We have obtained preliminary results showing that this occurs mainly because they target alternatively spliced transcripts with opposite modes of regulation. By grouping the probe sets targeting the same sets of transcripts and having the same neighbourhoods, it should be possible to construct transcript-oriented networks that are simpler, more complete and more reproducible [42].

To validate our method, we followed a widely used approach that assesses the quality of a particular list of genes by showing that the overrepresented GO terms characterising the genes belong to the same functional class [43]; this approach has been extensively applied to the clusters of genes found in coexpression networks [44,5,8]. To increase the validity of the test, we verified that the large regions we detected with the Markov Clustering Algorithm were not only characterised by a derivative of the "guilt by association" method [45] but were also conserved across four networks encompassing three evolutionarily close mammalian species. The extension of the study to four networks allowed us to add a test confirming the invariance of the local network topology by considering the reproducibility of the neighbourhood of a given gene across the four networks. The Keiko method that we developed – which enables the creation of 2D and 3D geometrical representations of the network while keeping the essential information encoded in CVM, with negatively and positively correlated probe sets being farther away or closer, respectively, according to the strength of the correlation – facilitates the visualisation and comparison of the networks. At the smallest scale, we observed that the local architecture is well conserved and is mainly shaped by strong positive correlations. With respect to the relationships between the regions defined at a higher scale, we observed that all four networks displayed a low mean inter-region connectivity. However, the strength and direction of the mean inter-region correlations were not conserved. We presume that this lack of reproducibility was mainly due to the lack of normalisation in the biological conditions used.

Several studies have used different species to construct coexpression networks [46], and it is interesting to see how our findings compare to their results. Among these studies, those aimed at delineating modules are not adapted for such comparisons. Modules are subsets of co-regulated genes associated with particular biological contexts – the subset of conditions under which co-expression takes place- and they form collections of numerous small overlapping lists of genes [47]. In contrast, our method partitions the entire gene set into a relatively small number of large, non-overlapping groups. J.M. Stuart et al. [8] searched for pairs of genes whose expression was significantly correlated in four evolutionarily diverse organisms (yeast, worms, flies and humans) in order to identify physiologically important, evolutionarily conserved examples of gene coregulation; they defined 12 components. As they did not list the metagenes belonging to each of the 12 components that they defined, we used the general biological description that they provided for each component and searched for regions with an overrepresentation of the GO terms linked to that description. Most of the correspondences were many-to-one relationships: components 2 (ribosome biogenesis), 5 (cell cycle), 6 (general transcription), 8 (translation initiation, elongation and termination, aminoacyl tRNA biosynthesis), and 9 (ribosomal protein subunits) corresponded to region 2 (nuclear processes); and components 10 (secretion) and 11 (neuronal) corresponded to region 5 (nervous system). Conversely, some components were split and gave rise to one-to-many relationships: component 3 had one function (energy generation, oxidative phosphorylation and TCA) linked to region 4 (energetic metabolism) and another function (gluconeogenesis) associated with region 6 (metabolism). Similarly, component 1 had one function (cellular cortex) linked to region 3 (adhesion) and another (signalling) associated with regions 1 (immunity), 2 (nucleus), and 3 (adhesion). Component 12 (lipid metabolism, peroxisome) corresponded to region 6 (metabolism), component 4 (proteasome) had no clear counterpart, and component 7 (animal specific) was too loosely defined to find any correspondence.

By applying principal component analysis (PCA) to the expression levels of orthologous human and mouse genes in different tissues, Chen et al. [44] identified 12 conserved gene expression response modes (CGEMs), each one characterized by a list of the most significant genes (around 70 genes). One of these specialized modes (protein biosynthesis and ribosome) corresponds to region 2 (nucleus). Five modes which are characterized by GO terms, Kegg Pathways, and COG classes corresponding to the same general function (e.g. immune response and Toll-like receptor signalling pathway (mode H13M12)) match in a one-to-one ratio to regions 1, 2, 3, 4, and 6. Five heterogeneous modes (e.g. cell adhesion and generation of energy (mode H10M8)) correspond to multiple regions, and one mode (organic acid transport, proton transport) has no particular match with any region. No mode has a clear match with the nervous system (region 5), although several nervous system tissues were present in the samples used.

Taking all of these studies together, we note that there were three types of potentially problematic results: first, there was a coexistence of groups with very general functions that corresponded to the six regions we defined (e.g. generation of energy, cell cycle) and groups with very narrow functions (e.g. gamma-hexachlorocyclohexane degradation, peroxisome); second, some separated groups had the same function (e.g. glycolysis/gluconeogenesis); and finally, some groups were heterogeneous (cell adhesion and transcription). We conclude that our method, when applied to inter-species comparisons, was capable of defining reproducible groups that had biological functions with the same degree of generality. Since our method generates networks with high mean connectivity, we assume that using high values of Pearson's correlation coefficient, e.g. higher than 0.6 or even 0.7, results in sparse networks (see Figure 2) which could explain the occurrence of very specialized groups and the splitting of otherwise homogenous large regions into several isolated groups when clustering methods are applied.

## Conclusion

The distinction between coexpression and covariation has never been clearly addressed with respect to the massive analysis of transcriptomic microarray data, and the choice between these two approaches has been mainly dictated by technical considerations: single- and dual-channel techniques results are de facto considered to be coexpression and covariation, respectively. Although several arguments, derived from large-scale studies on the reproducibility of inter-laboratory microarray results, converge toward the conclusion that variations (and more specifically lists of varying genes) are far more reproducible than are signals, it has only been in time course analyses that the benefits of the covariation approach have been recognized and single-channel results specifically manipulated to give covariation values [48,49]. The method that we have devised to calculate probe set correlation scores is the first to implement these principles, allowing the covariation approach to be applied to the major compendium of microarray results represented by Affymetrix data. Another advantage of our method is its ability to deal with circumstances in which the correlation between two given probe sets is positive in one comparison subset and negative in a second one. In such cases, the widely used Pearson's correlation coefficient is ineffective, while our method can properly calculate both positive and negative correlation scores.

We applied our method to four data sets covering three different mammalian species (humans, mice, and rats). In addition, we conducted in-depth comparisons between the corresponding covariation networks in order to validate our method. First, we determined that the four networks shared a similar network topology by measuring the probe set neighbourhoods. Second, we developed a technique to display the networks in 3D, revealing that the local assignation of the probe sets was conserved across the four networks. Finally, we applied the Markov clustering algorithm and showed that the transcriptional networks are organized into six loosely connected regions, each representing one of the main physiological functions in mammalian species and containing around 1000 genes.

## Methods

### Data Selection

#### Software Determination

To determine the version(s) of the software used to calculate the signals for each sample, we first searched in the corresponding GSM files for names specific to each version of the Affymetrix microarray suite. Mas3 was assigned if any of the terms "mas3", "mas 3", or "microarray suite 3" was found in the header. Mas4 and Mas5 were assigned on the same principles. The field names line was also scanned and the software version assumed to be Mas4 if in any of the fields "average difference", "pairs used", "avg diff", or "log avg" was encountered. The field "detection p-value" was considered indicative of the Mas5 version. In some cases, a second program was used to refine the signal values delivered by the Affymetrix software. For example, Dchip [39] utilisation was inferred if the names "dchip", "li-wong," or "li-wong" were present in the header. The names "rma" and "genespring," when found, indicated the use of the corresponding method or software [38,50].

#### Distance Between Samples

We constructed a median sample in which the rank of each probe set represented the median of its ranks over all the samples. We compared each sample with the median sample by applying the RDAM algorithm and selected all of the probe sets having a p-value smaller than, e.g., 0.005, in at least one comparison (see RDAM method, *infra*). We calculated the distance between each pair of experimental points in the probe set space, with log2 of the signal being used as the unit and with each axis representing one of the probe sets selected in the previous step (ranks were transformed into signals using an identical function for all the samples). Finally, we traced a dendrogram, a two-dimensional, "tree-like" diagram that summarizes the distances between experimental points, by connecting points according to their proximity.

#### Detection of Redundant Conditions

Median biological conditions were constructed by duplicating the median samples constructed as explained in the preceding sub-section. We compared each biological condition that survived the quality filtration steps with the median biological conditions by applying the RDAM algorithm, and selected in each comparison the varying probe sets at FDR 10% (see RDAM method, *infra*). We calculated the distance (d) between any two biological conditions by computing the number of common varying probe sets (#com) and by normalising by the minimum of varying probe sets in each of the two compared conditions (min(#var1,#var2)) as follows: d = 1-#com/min(#var1,#var2). The set of non-redundant conditions was constructed incrementally. The conditions that had the highest mean distances from all the other conditions were selected for seeding the process. Then the conditions, sorted from the highest mean distance to the lowest, were passed successively and added if (and only if) its distance from all the conditions selected in a previous step was greater than 0.20 (i.e. less than 80% similarity). Finally, a dendrogram was constructed to display the selected non-redundant conditions.

### Data analysis

#### RDAM method

Rank Difference Analysis of Microarray (RDAM) [30] allows the identification of statistically significant signal variations between two biological conditions. Each signal is first replaced by its rank in an ordered series of all the signals, and the rank is then scaled from 0–100. The scaled rank allows the definition of the expression level of each gene by placing its signal in the signal distribution; this simple transformation is in fact a normalisation method that makes all results directly comparable. Each pair of signal values for a given gene measured in two independent experiments is converted into a variation value by computing the difference in the corresponding ranks. To compensate for the fact that variation is dependent upon the rank value, the following local normalisation procedure is employed: *V*_*norm *_= (*V-m*_*v*_)/*s*_*v*_, where *m*_*V *_is the local mean value of the variation and *sV *is the local standard deviation of the variation, calculated by moving a window across the rank range. When applied to duplicated experiments, the standardized Rank Difference (zRD) is totally independent of the rank value. Therefore, all the points can be used to construct the empirical variation distribution in the case of the tested null hypothesis (no significant variation is expected when comparing biologically identical samples), allowing accurate and precise p-values to be assigned to the actually observed variation.

The following quantities were estimated by RDAM: the total variation (TV), the p-value of a variation, the false discovery rate (FDR), i.e., the percentage of false positives in a selection, and the sensitivity (S), i.e., the percentage of the total variation that is found in a given selection. Subsets of genes can be selected using any of the three latter parameters (p-value, FDR, or S) or a combination of them.

In the case of an absence of replicates, e.g. in comparisons made between each sample and the median sample to construct dendrograms (additional file 1), the empirical variation distribution observed between the test and the control retains the property of being largely independent of the rank and can be used to assign p-values.

If signal values are needed, for example to calculate a distance between the samples, a single function is used to convert all the ranks into signals. The function used is simply the observed relationship between the rank and the signal for a given sample.

### Architecture of Keiko maps

Probe sets located within a set of clusters covering a large portion of the Keiko map – e.g. clusters defined with Gene DIVER – were used to calculate the best projection plane defined by Principal Component Analysis (PCA). Each cluster is represented on this plane by a unique point located at the barycentre of all the probe sets of the cluster. For each probe set i of a cluster j, we calculate the mean positive  and negative  correlations of probe set i with all the other k probe sets of the cluster. The main trend of the relationships among the probe sets of the cluster is set to positive (or negative) if the mean  is greater (or smaller) than . The ratio  represents the strength of the positive (negative) correlation trend. Similarly, we define  or  between two clusters i and j. To display this information, we use Cytoscape, which allows the user to place points representative of clusters on the projection plane. Using this software, the main intra-cluster or inter-cluster correlation trends can be represented with red or blue lines, for positive or negative trends, respectively, whose widths are indexed on the trend strength.

### Generator

We use the GENERATOR tool [51] to group each set of co-varying genes into functionally analogous subsets. Such grouping allows an investigation into whether the set is comprised of several biological processes. GENERATOR takes two gene sets: the main set of genes to be analysed, and a background set that includes the other genes in the microarray. A clustering procedure is then applied to the main set to obtain functionally analogous subsets of genes. To cluster the genes, their associations with GO terms [52] are represented as binary data. As observed in Pehkonen et al. (2005), such data tends to be very high dimensional and to contain subgroups only in the small subsets of all the data attributes (GO terms). Thus, GENERATOR uses a clustering procedure based on Non-negative Matrix Factorization (NMF) [53-55]. NMF has shown good performance with sparse binary data and with large numbers of dimensions in text mining and in image analysis [56,54].

With the GENERATOR tool, the clustering of a gene set is performed several times for a particular number of clusters r. The solution with the smallest least squared error is considered to be representative of the cluster number r. The tool also performs clustering for different numbers of clusters from 1 (representing the gene set without grouping) to a user-selected number. Each clustering process is performed from a random starting initialization using NMF. A summary is then produced based on the different clustering solutions. There are two guidelines for interpreting such results (given in Pehkonen et al., 2005). First, when clusters remain similar in different clustering solutions, they can be interpreted as representing a non-random outcome. Second, the functional entities from broad to specific are observable in a set of clustering solutions from a small to a large number of clusters.

GENERATOR also shows an associated theme for each obtained cluster. This is performed by ranking GO terms within each cluster by their hypergeometric p-values, which compares the frequency of a given GO-term within a cluster against its frequency among the overall microarray. Additionally, GENERATOR removes GO terms with weak overrepresentation in the whole gene list (against the chip), as these can be considered non-important. Also, GO terms with weak overrepresentation in individual clusters (relative to other clustered genes) are removed, as they are not relevant to each obtained cluster.

## List of abbreviations used

CVM: covariation matrix; FDR: False Discovery Rate.

## Authors' contributions

JH developed the Keiko algorithm, imported and formatted the raw data, and improved the construction of the CVMs. PP adapted the GENERATOR algorithm to the needs of the study and participated in the functional analysis of the results. MB designed the study, developed the needed software, and wrote the paper. All authors read and approved the final manuscript.

## Supplementary Material

Additional file 1 ***Data processing and data filtering***. This PDF contains a comprehensive description of the whole data filtering process. Click here for file

Additional file 2 ***Information on experiments, biological conditions and samples***. This OpenOffice spreadsheet contains several tables describing the experiments, the biological conditions, and the experimental points used in this study. The table names indicate the chipset name (HS-U95 for the Human Genome U95 Set, HS-U133 for the Human Genome U133 Set, MM-U74 for the murine U74 Version 2, and RN-U34 for the Rat Genome U34 Set) and the type of information they contain (EXP for experiment, BIOL for biological condition, POINT for experimental point). Click here for file

Additional file 3 ***Conserved links***. This OpenOffice spreadsheet contains the mean percentages of conserved links when the neighbourhoods of two subsets of paired probe sets are compared. Click here for file

Additional file 4 ***Structure of the regions***. This OpenOffice spreadsheet contains the percentage of probe sets in each region, and the mean inter-region and intra-region strength and connectivity values. Click here for file

Additional file 5 ***Images of the CVM***. This PDF contains the images of the CVMs with their probe sets ordered by an independent clustering process inside each region, as explained in the legend to Figure 11. Click here for file

Additional file 6 ***Clustering with GENERATOR***. This OpenOffice spreadsheet contains several tables describing the genes contained in the six common regions found by the Markov clustering algorithm and in the clusters delineated by GENERATOR in each region. Click here for file
