# Supplementary material for: Construction and use of gene expression covariation matrix
Source: BMC Bioinformatics. 2009 Jul 13;10:214. doi: 10.1186/1471-2105-10-214 (PMC2720390; doi:10.1186/1471-2105-10-214)
Supplement: Additional file 1 — Data processing and data filtering. This PDF contains a comprehensive description of the whole data filtering process. [file 1471-2105-10-214-S1.pdf]

Data Filtering

We present here the data that we used, explain how the data was organized, and describe the quality filters that we developed to eliminate data that could potentially affect the construction of CVMs. The criteria we considered included the number of missing points, and we also introduced an expression level thresholding step that counts the number of points having the same signal value. The selection of pairs of replicates for each biological condition is a characteristic of our method, and care must be taken to use true biological replicates. We show how graphical techniques allowed us to detect suitable pairs of technical replicates by displaying either strong correlations between their ranks or an abnormally high number of variations in all the comparisons between the corresponding biological condition and all the other biological conditions. Most of the data used in this analysis were generated by the Mas4 or Mas5 algorithms, and we present a test that we performed to assess the potential effects of these algorithms on the results. Finally, we discuss the potential effects of sample redundancy and describe how we dealt with this problem.

1 - Selection of chipset Models and Downloading of Data

The method that we used to calculate CVMs relies on the ability to perform systematic comparisons between any two biological conditions present in sets of experiments coming from different laboratories. For this reason, not all technologies are suitable for this approach, which can only be applied if the signals from two different chips are directly comparable. Single channel technologies can allow such direct comparisons in two possible ways. First, the in situ probe synthesis technique that is used, for example, by Affymetrix [1] or NimbleGene[2], results in a nearly constant probe density across the surface of a chip. Second, density variations, which are inherent to the process of probe spotting, can be corrected using internal controls, as is done with the Applied BioSystem technology. Other technologies based on spotting, e.g. cDNA or oligonucleotide spotted arrays, must use double channel hybridization schemes with external controls to compensate for surface dissimilarities in probe density; because of this, they do not deliver reliable signal values that can be directly compared between different chips. Another important characteristic for our approach is the availability of datasets obtained over a large panel of experimental conditions with the same standardized collection of probes. These two criteria, plus others described in the Introduction, led us to restrict our analysis to datasets originating from Affymetrix platforms, and to consider four chipset models in which the number of biological conditions studied was greater than 100 as of October 2004 (see supplemental file exp-biol-point-info.ods). We organized the downloaded data into a structure in which the samples are grouped according to the biological conditions and experiments. With this nomenclature, experiments and samples correspond, respectively, to Geo Data Sets (GDS) and Geo Samples (GSM) in GEO. A given biological condition groups all the replicated experimental points (table 1, "before filtration" section).

chip set Model	before filtration			first filtration		end filtration		NR filtration	
	#exp	#biol	#point	#exp	#biol	#exp	#biol	#exp	#biol
Human Genome U95 Set	32	237	882	23	136	15	71	13	37
Human Genome U133 Set	24	174	538	19	137	18	126	13	88
Murine Genome U74 Version 2	56	351	808	50	251	37	205	36	89
Rat Genome U34 Set	22	183	543	18	119	13	96	13	41
	**134**	**945**	**2771**	**110**	**643**	**83**	**498**	**75**	**255**

Table 1 - Platform origin of the data downloaded from GEO.
For each model listed, the number of experiments (#exp), the number of biological conditions (#biol), and the number of samples (#points) are indicated. Columns labeled 'before filtration' indicate the raw figures at the time the data were downloaded. Columns labeled 'first filtration', 'end filtration,' and 'NR filtration' indicate the figures obtained once the unusable (first and end) and redundant samples (NR) were eliminated. For these three groups, the number of samples is twice the number of biological conditions.

2 - Characterisation and Validation of Samples

A preliminary step in RDAM is the transformation of each signal into a relative rank, i.e. the placing of the signal value into the signal distribution, expressed on a scale of 0-100. This transformation is important because it acts as a normalisation procedure that allows different samples to be compared. However, if some probe sets are missing, this transformation will result in a systematic bias towards higher ranks, which could affect the RDAM analysis. To minimize this possibility, we decided to eliminate all samples having more than 500 missing probe sets. Figure 1 indicates the number of missing probe sets in the U95 dataset. Although no samples were eliminated for this chipset model, we note that experiment 18 had a high number of missing probe sets (441). This experiment was analyzed with DChip, and it is possible that these probe sets were dropped during this step for an unknown reason. For all the other samples, the number of missing probe sets was less than 100 and corresponded to dispensable probe sets like spikes and controls. All of these missing probe sets have had their signals set equal to the minimum of the existing signals.

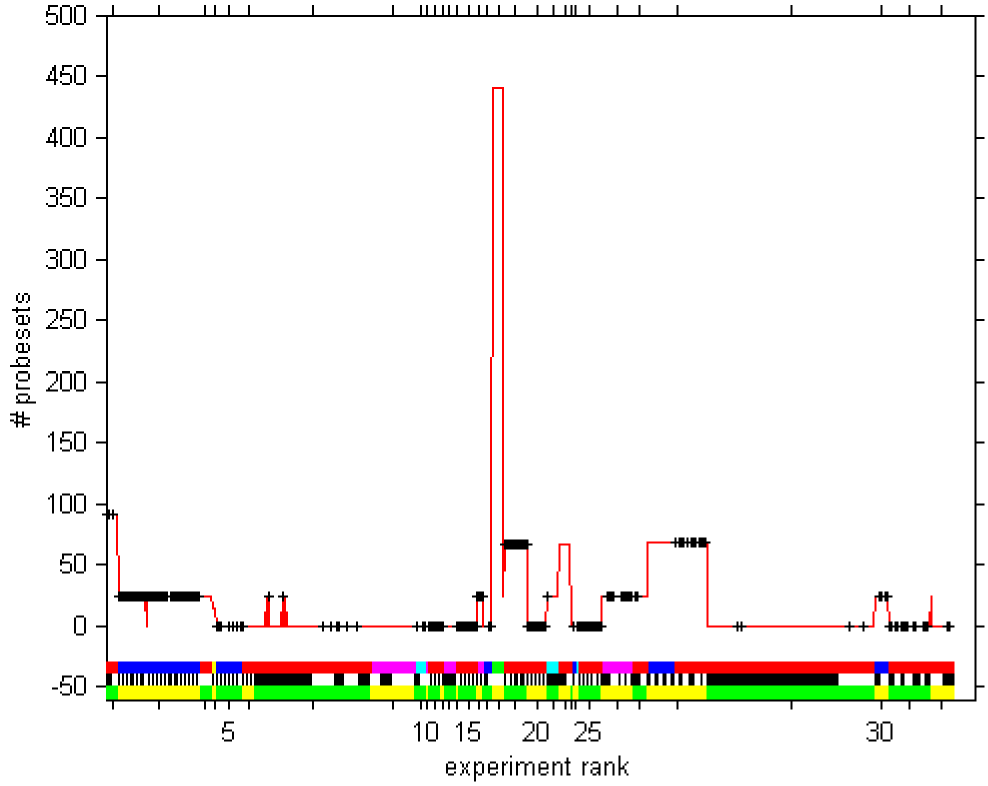

Figure 1 – Number of missing probe sets in the U95 samples.
In red is plotted the number of missing probe sets in the 882 samples of U95.Ticks on the X axis indicate the experiment rank. Samples are ordered by biological condition within each experiment. Black crosses mark the two samples per biological condition that have been conserved, at the end of all the controls, for the construction of the CVM. At the bottom of the figure, three coloured bars indicate the respective correspondence of the samples to the experiments (alternation of green and yellow), biological conditions (alternation of black and white), MAS5 (red or magenta) or MAS4 (blue or cyan) analysis, and the performance of a possible supplementary analysis step, e.g. DChip in green and RMA in yellow. Assignation to MAS4 or MAS5 was based either on the information found in the description of the data (blue and red) or the presence/absence of negative signals (cyan and magenta).

Another important potential source of bias is the truncation of the data, which generally results from the application of a threshold at some step in the data processing. The consequence of this is that numerous different probe sets have identical signal values, causing the true order of the probe sets to be lost and leading to the random assignation of their ranks. To identify this potential problem, we sorted the signal values and searched for the longest run of identical values. Figure 2, which displays the size of the longest run (red line), shows that the entire experiment 8, and around half of experiments 6 and 28, had runs of more than 500 in number, which led us to filter out the corresponding samples. In the same figure, we have plotted the total number of different signal values used in the sample (blue line). This quantity is related to the numerical precision of the measurement, i.e., the number of significant digits employed. If this numerical precision is high, then all measurements will be different, as is the case, exceptionally, in experiment 18. Conversely, a very low level of numerical precision will result in numerous runs having identical values and will thus drastically decrease the diversity of the signal values; this is exemplified by experiment 6, in which the 12,625 probe sets of the U95 chip A are represented by only about 400 different values. However, it is worth noting that even with such a low level of precision, the effect on the RDAM analysis will be negligible, because the random assignation of rank will only occur over a very

small range: the mean number of probe sets having the same signal in this case will be around 30 (for a run of 100 probe sets having exactly the same signal, the rank would vary between r and r±0,79, a difference which would only exceptionally be statistically significant). Consequently, we did not use this information to filter out any samples.

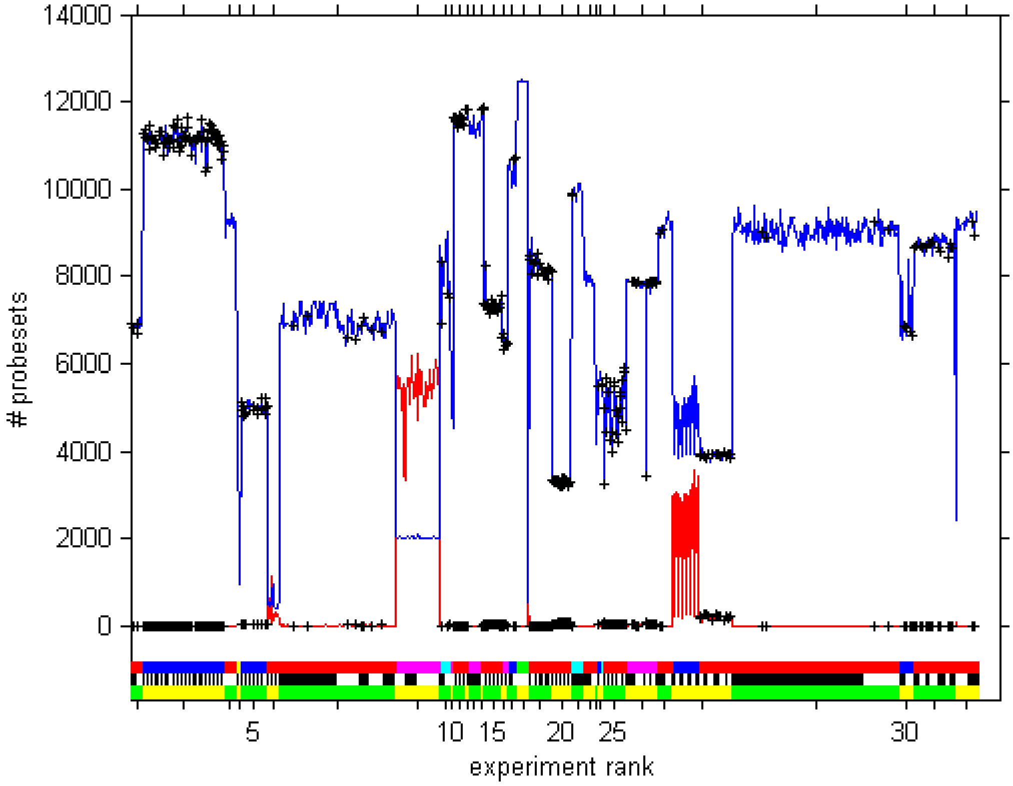

Figure 2 – Size of the longest run and precision of the signal measure in the U95 samples. *The size of the longest run, i.e. the largest subset of probe sets that all have the same signal value, is plotted in red. The total number of different signal values used is plotted in blue. See the legend to figure 1 for additional information about the meanings of the different symbols used.*

The downloaded data were initially processed either with MAS4 or MAS5, the two main versions of the Affymetrix analysis suite currently available. With respect to our method, the most important difference between these two versions is the way in which they calculate the single compound signal of a probe set from several measured probe signals (around 20 per probe set): MAS4 can generate negative numbers, whereas MAS5 only produces positive numbers. As the completion of this field is not mandatory in GEO, we searched for the relevant information in the headers of the GSM files, as explained in the Methods section. On the basis of these incomplete pieces of information, we first tentatively assigned a MAS version number to 26 experiments out of 32. In order to confirm this tentative assignation and to complete the classification, we plotted (figure 3) the minimal signal values (blue line). The occurrence of numerous negative values in experiment 21 suggests that their initial classification as MAS5-processed is erroneous. The absence of negative signals in experiment 28, which was classified as MAS4, is explained by the application of a threshold for the first half of the samples. Experiment 13, which was classified as MAS4, was changed to MAS5 because no negative signals were present. Experiments not classified in the first step were designated MAS4 for experiments 9 and 24 and MAS5 for experiments 8, 10, 16 and 26. Experiment 18 was analyzed by dChip and experiment 4 by RMA.

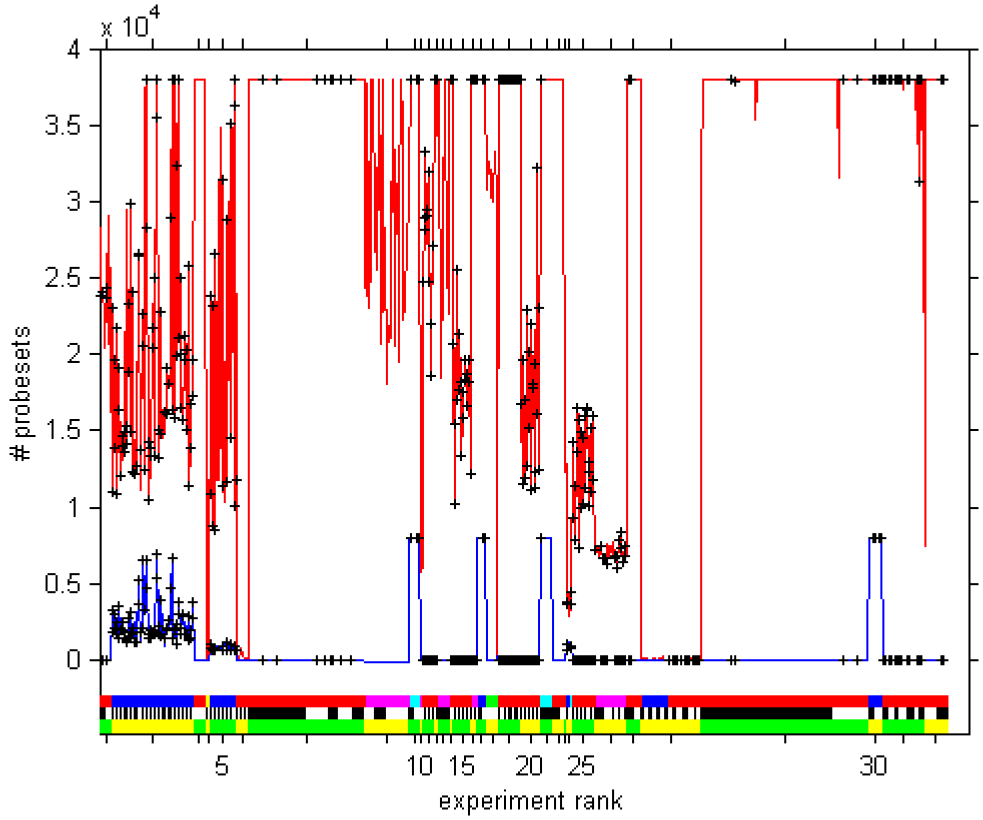

Figure 3 – Minimal and maximal signal values in the U95 samples.
The maximal signal value is plotted in red (values are truncated at 38,000). The opposite of the minimal signal value is plotted in blue (values are truncated at -8000). See figure 1 legend for additional information about the meanings of the different symbols used.

3 - Selection of Replicates

We performed the multiple biological condition comparisons by applying the Rank Difference Analysis of Microarray (RDAM) method [3][4]. As RDAM can take advantage of duplicated measurements to evaluate the noise distribution and to improve statistical power, we did not consider biological conditions represented by only one sample, and we selected exactly two replicates for all of the other biological conditions. We calculated the distance between any two samples (see Methods) and traced a dendrogram. This graphical representation of the whole dataset allowed us to identify the two most similar replicates for each condition, to filter out questionable samples, and to visualize the quality of the entire dataset. We show in figure 4 some branches of the U95 dendrogram. Panel A displays some samples whose names contain the character @ to indicate that the corresponding biological condition was not replicated and therefore not used (see also sample 27 in panel E). In panel B, the null distance between the pairs of samples (125,137) and (122,124) in experiment 5 means that the same sample has been used twice under two different names. We encounter the same situation in panel C, where the same sample was used in two different experiments. Panels A, C and E show that similar biological conditions are grouped. It is worth noting that samples of panels A and C, both of which pertain to muscle, are grouped separately according to their pathological or physiological origins. Such groupings of similar biological conditions independently of their experimental source is not systematic, and experiments 2 (panel E) and 25 (panel D) show a situation in which the experimental origin had a greater weight than the biological condition in the grouping process.

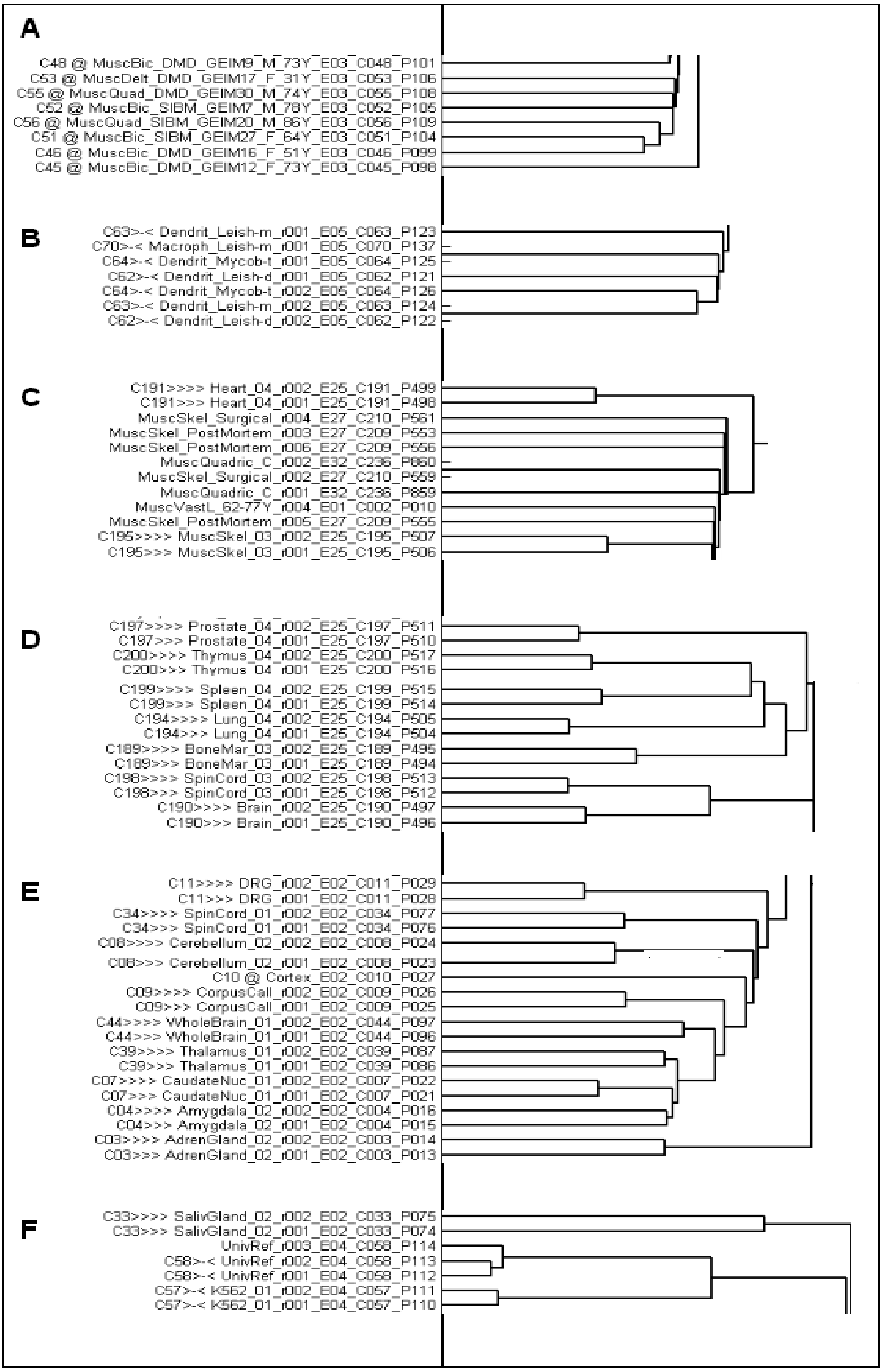
A
C48 @ MuscBic_DMD_GEIM9_M_73Y_E03_C048_P101
C53 @ MuscDelt_DMD_GEIM17_F_31Y_E03_C053_P106
C55 @ MuscQuad_DMD_GEIM30_M_74Y_E03_C055_P108
C52 @ MuscBic_SIBM_GEIM7_M_78Y_E03_C052_P105
C56 @ MuscQuad_SIBM_GEIM20_M_86Y_E03_C056_P109
C51 @ MuscBic_SIBM_GEIM27_F_64Y_E03_C051_P104
C46 @ MuscBic_DMD_GEIM16_F_51Y_E03_C046_P099
C45 @ MuscBic_DMD_GEIM12_F_73Y_E03_C045_P098
B
C63>-< Dendrit_Leish-m_r001_E05_C063_P123
C70>-< Macroph_Leish-m_r001_E05_C070_P137
C64>-< Dendrit_Mycob-t_r001_E05_C064_P125
C62>-< Dendrit_Leish-d_r001_E05_C062_P121
C64>-< Dendrit_Mycob-t_r002_E05_C064_P126
C63>-< Dendrit_Leish-m_r002_E05_C063_P124
C62>-< Dendrit_Leish-d_r002_E05_C062_P122
C
C191>>>> Heart_04_r002_E25_C191_P499
C191>>> Heart_04_r001_E25_C191_P498
MuscSkel_Surgical_r004_E27_C210_P561
MuscSkel_PostMortem_r003_E27_C209_P553
MuscSkel_PostMortem_r006_E27_C209_P556
MuscQuadric_C_r002_E32_C236_P860
MuscSkel_Surgical_r002_E27_C210_P559
MuscQuadric_C_r001_E32_C236_P859
MuscVastL_62-77Y_r004_E01_C002_P010
MuscSkel_PostMortem_r005_E27_C209_P555
C195>>>> MuscSkel_03_r002_E25_C195_P507
C195>>> MuscSkel_03_r001_E25_C195_P506
D
C197>>>> Prostate_04_r002_E25_C197_P511
C197>>> Prostate_04_r001_E25_C197_P510
C200>>>> Thymus_04_r002_E25_C200_P517
C200>>> Thymus_04_r001_E25_C200_P516
C199>>>> Spleen_04_r002_E25_C199_P515
C199>>> Spleen_04_r001_E25_C199_P514
C194>>>> Lung_04_r002_E25_C194_P505
C194>>> Lung_04_r001_E25_C194_P504
C189>>>> BoneMar_03_r002_E25_C189_P495
C189>>> BoneMar_03_r001_E25_C189_P494
C198>>>> SpinCord_03_r002_E25_C198_P513
C198>>> SpinCord_03_r001_E25_C198_P512
C190>>>> Brain_r002_E25_C190_P497
C190>>> Brain_r001_E25_C190_P496
E
C11>>>> DRG_r002_E02_C011_P029
C11>>> DRG_r001_E02_C011_P028
C34>>>> SpinCord_01_r002_E02_C034_P077
C34>>> SpinCord_01_r001_E02_C034_P076
C08>>>> Cerebellum_02_r002_E02_C008_P024
C08>>> Cerebellum_02_r001_E02_C008_P023
C10 @ Cortex_E02_C010_P027
C09>>>> CorpusCall_r002_E02_C009_P026
C09>>> CorpusCall_r001_E02_C009_P025
C44>>>> WholeBrain_01_r002_E02_C044_P097
C44>>> WholeBrain_01_r001_E02_C044_P096
C39>>>> Thalamus_01_r002_E02_C039_P087
C39>>> Thalamus_01_r001_E02_C039_P086
C07>>>> CaudateNuc_01_r002_E02_C007_P022
C07>>> CaudateNuc_01_r001_E02_C007_P021
C04>>>> Amygdala_02_r002_E02_C004_P016
C04>>> Amygdala_02_r001_E02_C004_P015
C03>>>> AdrenGland_02_r002_E02_C003_P014
C03>>> AdrenGland_02_r001_E02_C003_P013
F
C33>>>> SalivGland_02_r002_E02_C033_P075
C33>>> SalivGland_02_r001_E02_C033_P074
UnivRef_r003_E04_C058_P114
C58>-< UnivRef_r002_E04_C058_P113
C58>-< UnivRef_r001_E04_C058_P112
C57>-< K562_01_r002_E04_C057_P111
C57>-< K562_01_r001_E04_C057_P110

Figure 4 – Selected portion of the dendrogram of the U95 samples.

The samples are named as follows: a descriptive part is constructed by concatenating information recovered from the GDS files (for example Dendrit_Leish-m is the concatenation of the cell type information (dendritic cells) and the name of an infecting pathogen, Leishmania major). This name may be followed by the rank of the replicate (e.g. r001,r002). At the end are indicated the rank of the experiment, the rank of the biological condition, and the rank of the sample, preceded respectively by the letters E, C, and P. Other information and the decision drawn from the dendrogram processing is indicated before this composite name. The condition rank is recalled, followed by a symbol indicating, e.g., that the sample is used as a member of the duplicates representing the biological condition (>>>>), or that it has been discarded because of an absence of duplicates (@) or for some other reason, as indicated in the text (>-<). All panels use the same scale to represent distances.

In a first step, the two most similar duplicates were selected on the condition that they were distinct. We then inspected the scatter plots of the ranks of duplicates and found that three classes could be easily defined, as exemplified in figure 5. Most of the duplicates were similar to those shown in panels B and C and were considered adequate. Some duplicates were clearly of poor quality, such as those displayed in panel D, and the corresponding conditions-- i.e.the entire experiment 6 of the U95 dataset-- were not used. Conversely, some duplicates were abnormally similar, e.g., as shown in panel A, and were discarded as well because they were technical replicates (visible on panel F of figure 4). There were also some intermediate cases between those shown in panels A and B for which it was difficult to determine if they were technical replicates. These cases were resolved by visual inspection of comparison heat maps (see figures 8 and 10).

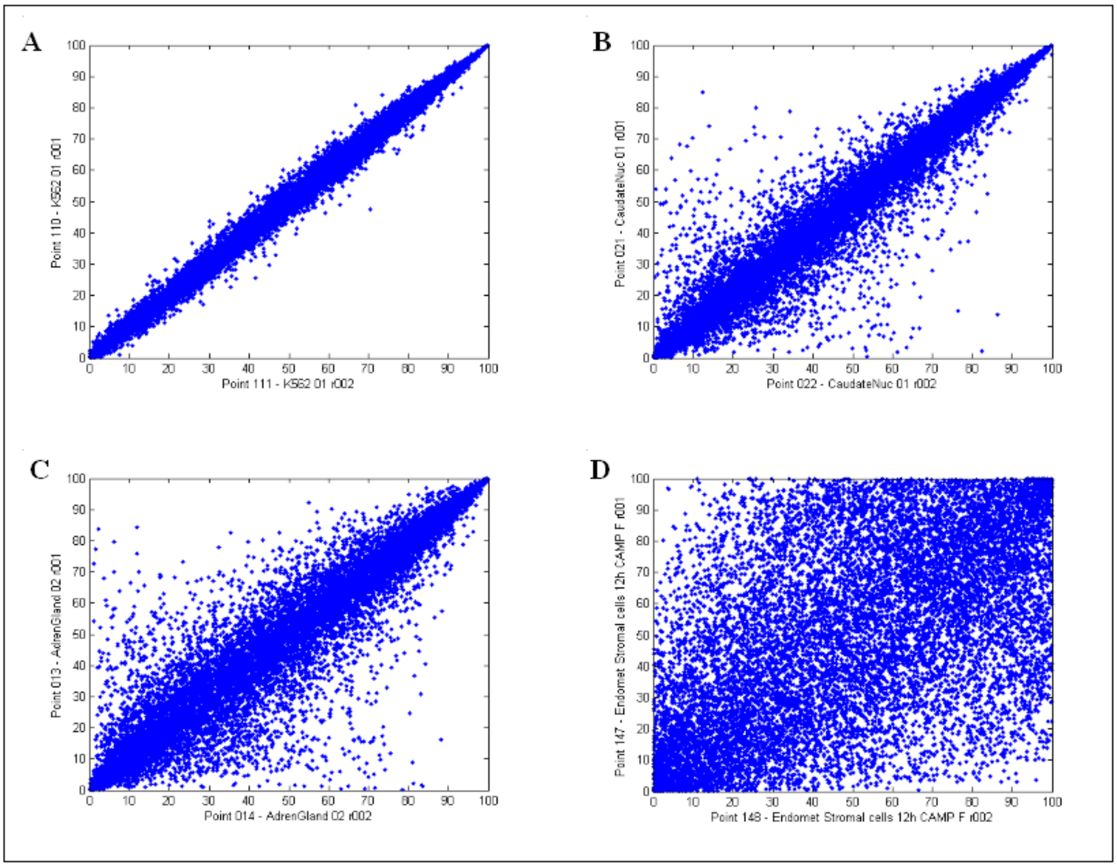

Figure 5 – Scatter plots of ranks define three classes of duplicates
Ranks of one duplicate are plotted against the ranks of the other duplicate.

Following the application of these successive control steps, 101 biological conditions out of 237 were eliminated from the U95 dataset. The discarded conditions corresponded to the following: 2 not measured with chip A, 70 without replicates, 12 with a longest run of greater than 500, 11 with the same sample used twice, 4 with poor reproducibility, and 2 with technical replicates. Table 1 summarizes the effects of this first filtration step on the four chipset models used in this study: 18% of the experiments (24/134) and 32% of the biological conditions (302/945) were eliminated.

4 - Rank Assignation for Data Analyzed by MAS4

The RDAM method was initially applied to data processed by MAS5, and all signals were given a different rank. Unlike with MAS5, however, the algorithm used in the MAS4 version of the Affymetrix software can give negative values when calculating the compound signals of particular probe sets. Because negative values cannot be used as a measure of transcript concentration, it might be tempting to assign a rank of zero to all negative signals. Another possibility would be to rank all values, irrespective of their sign, which would have the advantage of allowing MAS4 and MAS5 data to be treated similarly. We tested both of these ranking schemes on biological conditions analyzed with MAS4, and found that there was little difference between the CVMs constructed from the two. Figure 6 shows that in the CVMs constructed either by using a different rank for each signal value or by setting the rank to zero for negative signals, all probe sets have roughly the same connectivity degree. We thus concluded that the ranking scheme used for MAS5 data could be applied to MAS4 data as well.

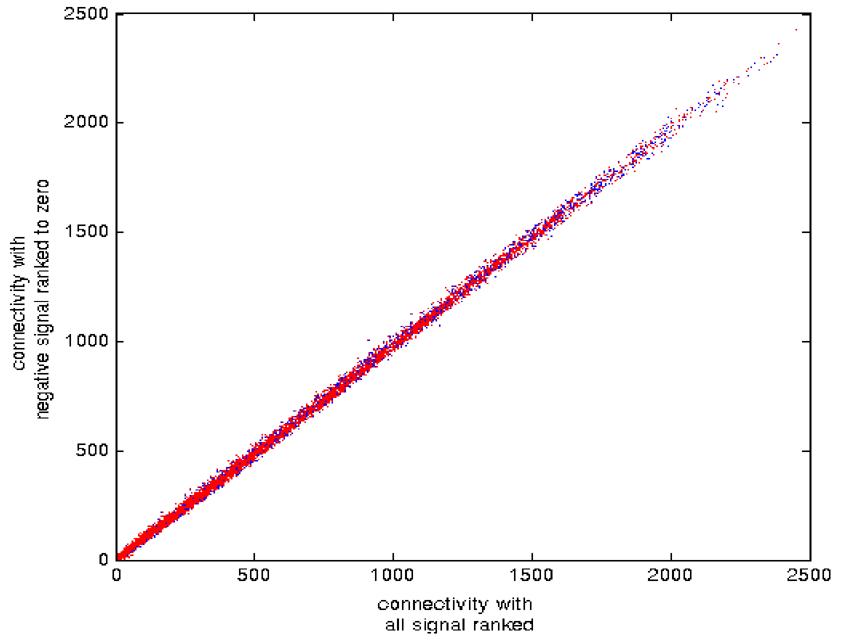

Figure 6 – Similarity in connectivity degree between CVMs constructed using two types of ranking applied to biological conditions analyzed with MAS4 in U95 experiments.
For a particular probe set, the connectivity degree represents its number of neighbours, i.e. the number of probe sets that have a correlation with it. In red are plotted the connectivity degrees between the two CVMs with positively correlated values, and in blue are the connectivity degrees between the two CVMs with negatively correlated values.

5 - MAS4 vs MAS5 Comparisons

Another point of concern is the compatibility of MAS4 and MAS5 results. It is worth noting that the samples belonging to the ten biological conditions common to experiments 2 (GSE96) and 25 (GSE803) form two disjoint groups in the dendrogram (figure 4). This absence of reproducibility could reflect either a systematic artefactual bias in one of the experiments or the existence of differences between the types of results. While the first explanation cannot be ruled out, an analysis of an experiment [5] in which the samples were treated with the two versions of the MAS software demonstrated that the type of result had a predominant effect, as shown in figure 7. It must be stressed that in this particular experiment the samples were scanned using the settings recommended by Affymetrix for the MAS4 version (photon multiplier tube (PMT) set at 100%). The drastic effect observed in figure 7 results therefore from the changes in the algorithm. In addition, we observed that the use of the MAS5 algorithm results in a dendrogram that groups samples belonging to the same biological condition, whereas they are mixed in dendrograms constructed from results analyzed by the MAS4 algorithm. It therefore seems undesirable to mix MAS4 and MAS5 results, because numerous statistically significant variations could be present between identical biological conditions processed by the two methods. It would thus be preferable to exclusively consider MAS5 results.

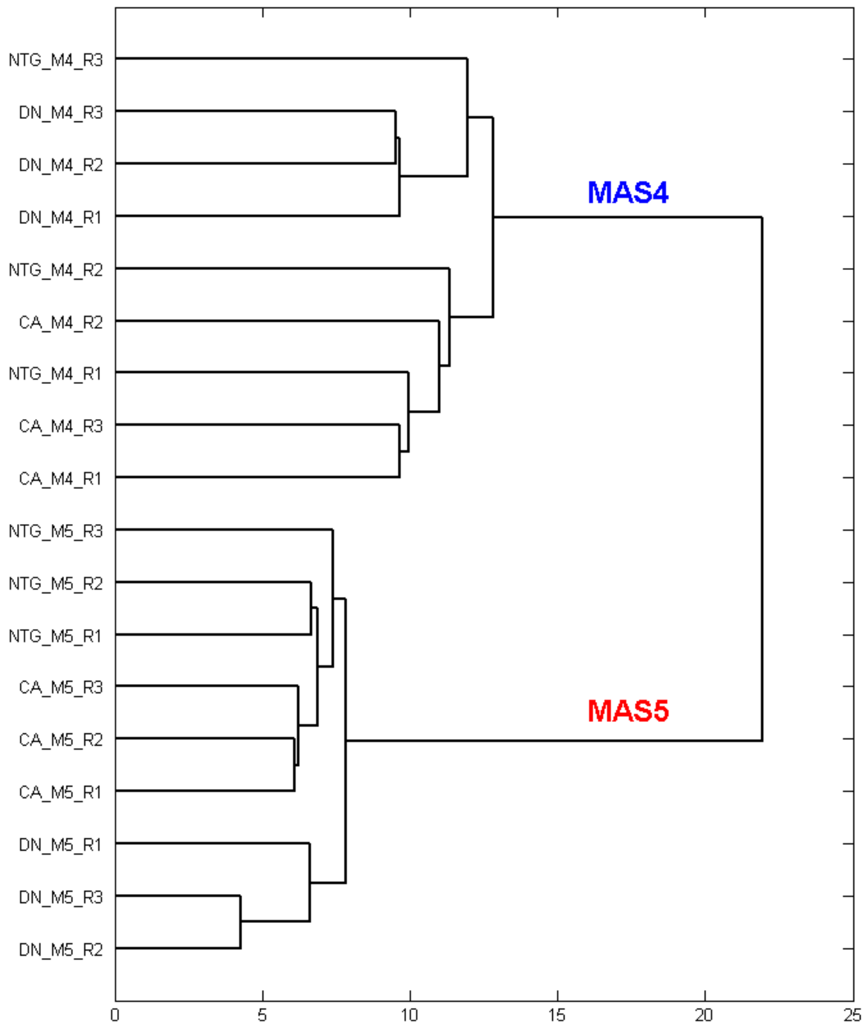

Figure 7 – Effect of MAS software version.
Samples (GSE558 [6]) are named by concatenation of the biological description, MAS version

number, and replicate rank: NTG: Non-transgenic, DN: Dominant-negative PI3 kinase, CA: Constitutively active PI3 kinase.The dendrogram was constructed in the same way as that displayed in figure 4 (see the 'distance between samples' section in the Methods).

6 - Comparisons between Biological Conditions

Following the first filtration step, we performed systematic comparisons between every pair of biological conditions, e.g. 136*135/2=9180 comparisons for the U95 chip set model (see column 'first filtration - #biol' of table 1). A graphical representation of these comparisons was used to display their general characteristics. An inspection of panel A of figure 8, which shows the estimated total variation for the increased and decreased probe sets in each comparison, shows that intra-experiment comparisons detected far less variation than those carried out between experiments. This property, which results in the presence of dark squares all along the main diagonal, reflects the tight relationships that exist between the samples from these experiments (see, for example, experiments 3, 4, 6, 9, 12, 13, and 17). Experiments 2 and 16 deviate from this rule because they involve a panel of different tissues. The pitfall that can be encountered when combinations of Mas4 and Mas5 analyses are used is evidenced by the presence of four contrasted patches: two darker patches (experiments 1 to 5 vs experiments 1 to 5, and experiments 6 to 18 vs experiments 6 to 18) and two lighter patches (experiments 1 to 5 vs experiments 6 to 18, and experiments 6 to 18 vs experiments 1 to 5). As most of the samples corresponding to the dark patches are either of the Mas4 (experiments 2, 3 and 5 for the first patch) or Mas5 type (experiments 6, 7, 8, 9, 10, 12, 13, 14, 16, 17 and 18 for the second patch), it must be concluded that comparisons between Mas4 and Mas5 samples will generally detect spurious variations. These observations led us to eliminate Mas4 samples from our systematic comparisons.

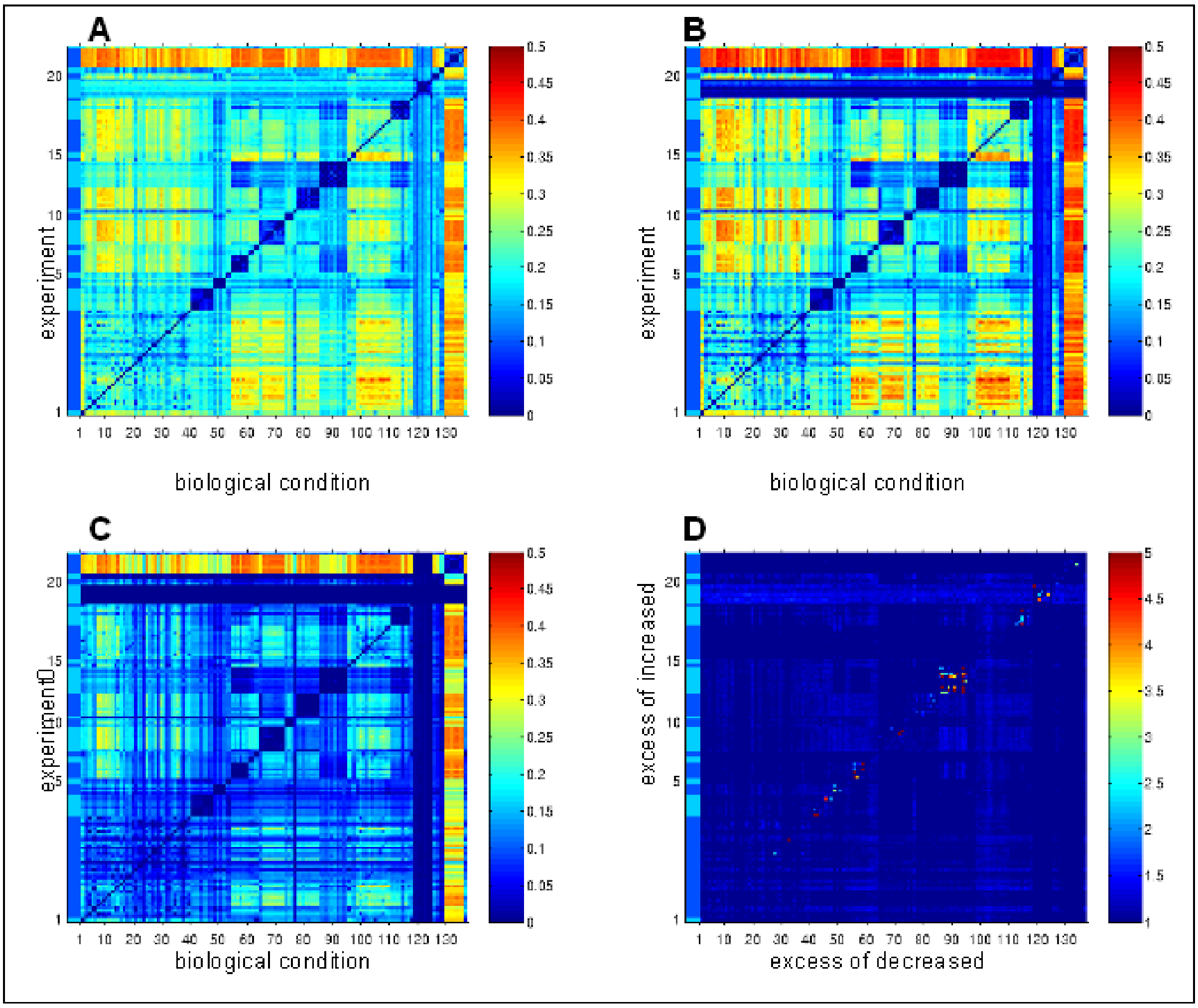

Figure 8 – Graphical display of the 9180 systematic comparisons made in the HG-U95 dataset. *The rank of the biological condition is plotted on the abscissa and the rank of the experiment on the ordinate. Since some experiments have been eliminated, the numbering of the experiments differs from that used in figures 1, 2 and 3. For example, the experiments containing Mas4 samples that were numbered 2, 5, 9, 17, 21, 24 and 30 in the earlier figures are numbered 2, 3, 5, 11, 14, 15 and 21 here. Experiment 19 (28 in figure 1) does not contain any Mas4 samples in this figure (eliminated in the first filtration step). For panels **A**, **B** and **C**, the coordinates (i,j) where i is smaller than j indicate the number of over-expressed probe sets in the comparison i vs j. The coordinates (j,i) can be interpreted either as the number of over-expressed probe sets in the comparison j vs i or as the number of under-expressed probe sets in the comparison i vs j. Coordinates are expressed as (line,column), and the coordinate (0,0) is at the bottom left corner. A colour bar on the right of each panel indicates the range of the heat map used.*
A- *Estimates of the total variation expressed as a fraction of the total number of probe sets (12,625 in the HG-U95 chip set model).* ***B-*** *Fraction of probe sets considered significant at the level of 10% FDR,*
C- *Fraction of probe sets considered significant at the level of 1% FDR,*
D- *Excess of one type of variation: in the comparison i vs j, if the estimated total variation for the increased probe sets is larger than the estimated variation for the decreased probe sets, then the ratio increased:decreased is plotted in (i,j), and (j,i) is set to one. Conversely, if the estimated total variation for the increased probe sets is lower than the estimated variation for the decreased probe sets, then the ratio decreased:increased is plotted in (j,i), and (i,j) is set to one.*

An inspection of the comparison heat maps revealed that experiments 19 and 22 were abnormal, which had not been detected in the previous quality filtering steps. In all of the

comparisons involving the biological conditions of experiment 19, virtually no probe sets varied at the level of FDR 10% (panel B); this could be partly explained by the poor reproducibility between replicates for this experiment. Inversely, in all the comparisons involving the biological conditions of experiment 22, at least 60% of the probe sets varied. As shown in figure 9, this abnormal outcome is probably due to an error in the assignation of signals to the probe sets, resulting in a randomization of the signal values in the GSM files.

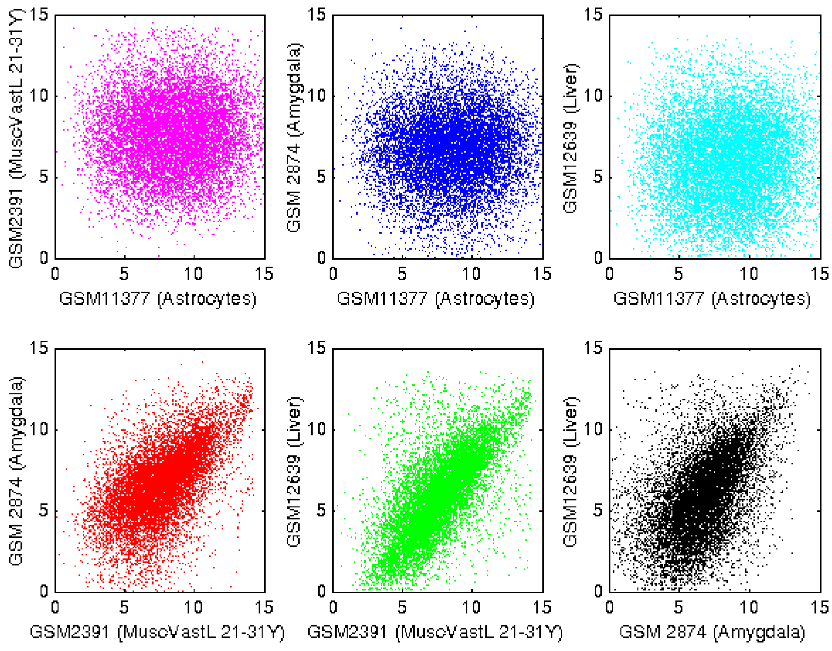

Figure 9 – Comparison of GSM11377 (GSE758) with three different tissues.
In the upper part of the figure, the log2 of the signals for three different tissues is plotted against the log2 of the signals of the GSM11377 sample, which belongs to experiment 22 of figure 8. The distribution of points over the circle indicates that GSM11377 is not correlated with any of the other three conditions. In contrast, when the three conditions are compared two by two (in the lower part of the figure), there is always some correlation in spite of their differing origins (amygdala, liver, and muscle).

It is sometimes difficult to determine by a simple visual inspection that two replicates from a given biological condition are technical replicates because their rank plot is intermediate between categories A and B of figure 5. In such cases, the inspection of comparison heat maps can help to confirm this status. For example, figure 10 shows that in the comparison heat map of chipset MG-U74, experiments 8 (GSE 369), 44 (GSE934) and 46 (GSE986) display a systematically high level of variation with respect to all other conditions and should therefore be considered as technical replicates. However, experiment 37 (GSE700), which has a similar pattern, does not seem to have technical replicates, as judged from its rank plots.

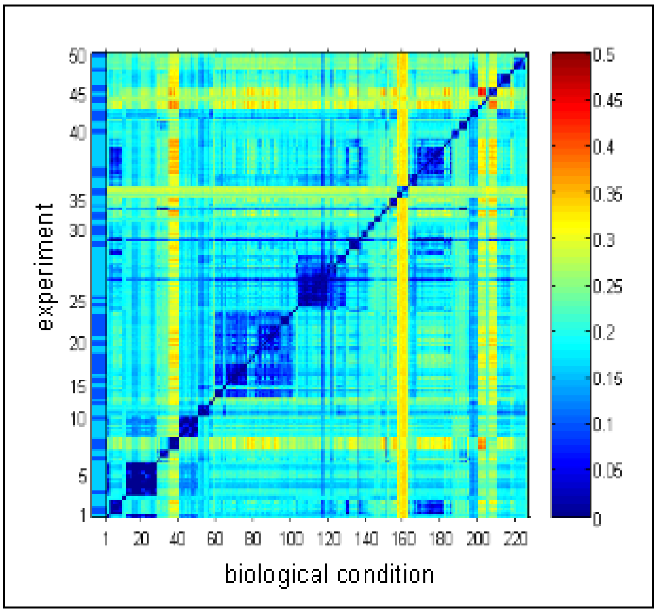

Figure 10 – Effect of technical replicates in the MG-U75 dataset.
Technical replicates (experiments 8, 44, and 46) are characterized by a specific signature on a heat map displaying the total number of variations estimated for all the comparisons. The resulting pattern is a constant, high level of variation for all the comparisons, reflected by the presence of horizontal and vertical lines with warmer colours than the general background.

Table 1 summarizes the effect of this final filtration step on the four chipset models used for this study: 38% of the experiments (51/134) and 47% of the biological conditions (447/945) were eliminated.

7 - Characteristics of Comparisons

Having selected all the suitable biological conditions, we next performed systematic comparisons between pairs of conditions by applying the RDAM method (see in Methods). For example, the 71 biological conditions conserved in the U95 dataset out of the 237 initially present gave 71*70/2=2485 comparisons. Figure 11 displays the results of these comparisons for the four chipset models used in this study. It can be concluded from the evenly distributed values of the fraction of varying probe sets that the two filtration steps we applied were successful in identifying biological conditions that do not produce abnormal patterns in this type of representation. Table 3 presents certain statistical properties of the comparisons performed in the four chipset models used. The first conclusion is that a very high and rather constant mean selection yield is observed: 78% to 89% of the estimated total variation is retained when a selection at FDR 10% is applied. The second conclusion is that the mean total variation is very high as well (17%, 14%, 18% and 16%, i.e. 2150, 3110, 2250 and 1400 varying probe sets in each comparison, respectively).

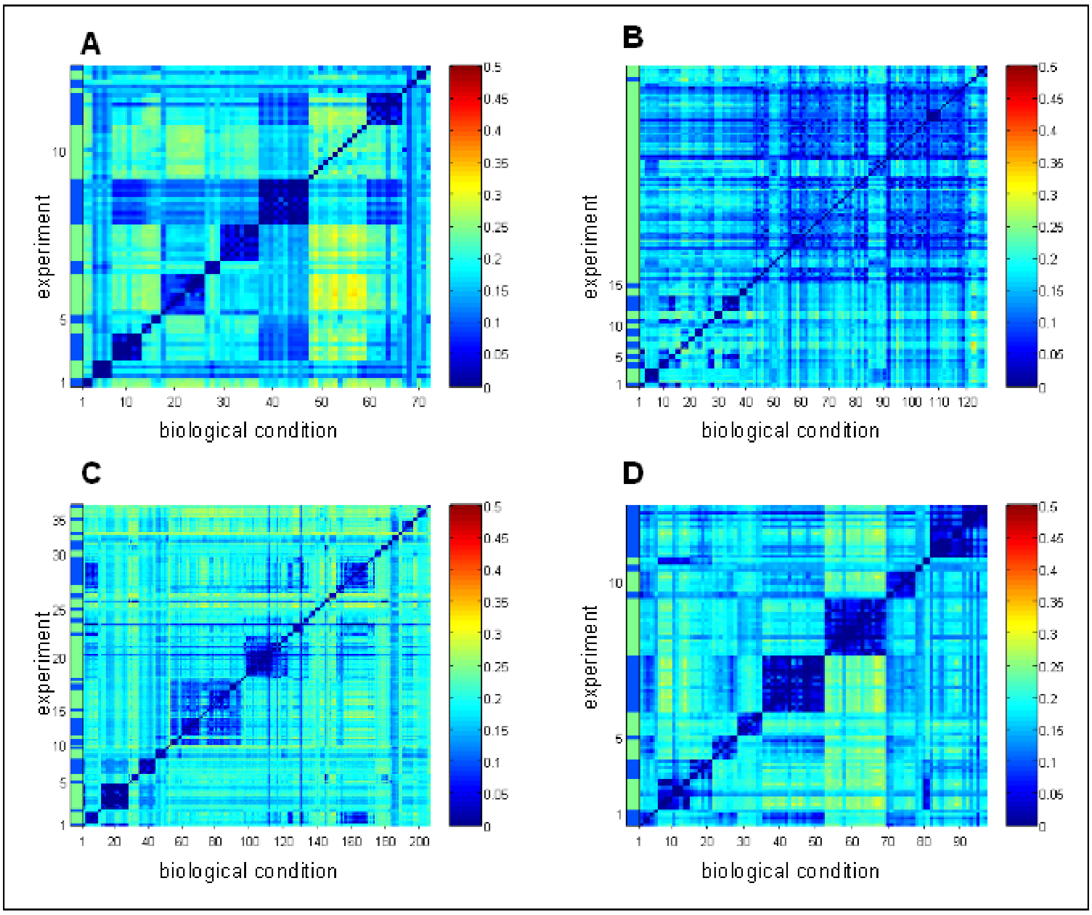

Figure 11 - Graphical display of the total variation present in all the comparisons made in each of the four datasets (HG-U95, HG-U133, MG-U74 and RG-U34).

In these plots, the biological conditions are displayed that were analyzed by MAS5 and that passed all the quality filters. No "outlier" pattern is visible, as is the case in figures 7 and 10.

chipset	# biol	# comp	s100%	fdr 10%	fdr 1%	fdr at s 50%	s at fdr 10%	s at fdr 1%
Human Genome U95 Set (12,625 ps)	71	2,485	0.16 ±0.06	0.15 ±0.09	0.09 ±0.06	0.06 ±0.11	0.74 ±0.31	0.45 ±0.27
Human Genome U133 Set (22,283 ps)	126	7,875	0.14 ±0.05	0.13 ±0.06	0.07 ±0.04	0.04 ±0.07	0.77 ±0.20	0.43 ±0.19
Murine Genome U74 Version 2 (12,488 ps)	205	20,910	0.18 ±0.05	0.19 ±0.06	0.11 ±0.05	0.02 ±0.07	0.88 ±0.17	0.57 ±0.18
Rat Genome U34 Set (8,799 ps)	96	4,560	0.16 ±0.06	0.15 ±0.07	0.08 ±0.05	0.04 ±0.11	0.79 ±0.25	0.46 ±0.21

Table 2 - Statistical properties of comparisons
For each of the models listed, the table presents the number of biological conditions (#biol), the number of comparisons made (#comp), and the mean proportion of genes selected in three conditions: 100% of sensitivity (s100%), 10% of FDR (fdr10%), 1% of FDR (fdr1%), the mean FDR at 50% of sensitivity (fdr at s50%), and the mean sensitivity at FDR 10% (s at fdr 10%) and 1% (s at fdr 1%). Mean values are followed by the corresponding standard deviations.

8 - Detection of Redundant Biological Conditions

Another potential source of bias in the construction of CVMs is the presence of multiple experiments that have different numbers of similar biological conditions. If an experiment contains many similar biological conditions, as is the case, for example, with GSE 314 (experiment 5 of figure 10, with 16 similar conditions), then most of the probe sets varying in a given comparison between one condition from this first experiment and a second condition from another experiment should also vary in comparisons between other similar conditions from the first experiment and the used second condition. For other, smaller experiments, e.g. GSE363 (experiment 7 of figure 10, with 4 similar conditions), the effect will also be present but to a smaller extent. It is therefore important to give the same weight to each biological condition and to eliminate redundant conditions. We constructed a distance matrix and defined as redundant those conditions that shared more than 80% identity (see figure 12 and the 'detection of redundant conditions' section of the Methods). After this process, the number of conditions in chipset models HG-U95, HG-U133, MG-U74, and RG-U34 was, respectively, 37, 84, 89, and 41 (versus 71, 126, 205, and 96 at the completion of the quality filtering steps).

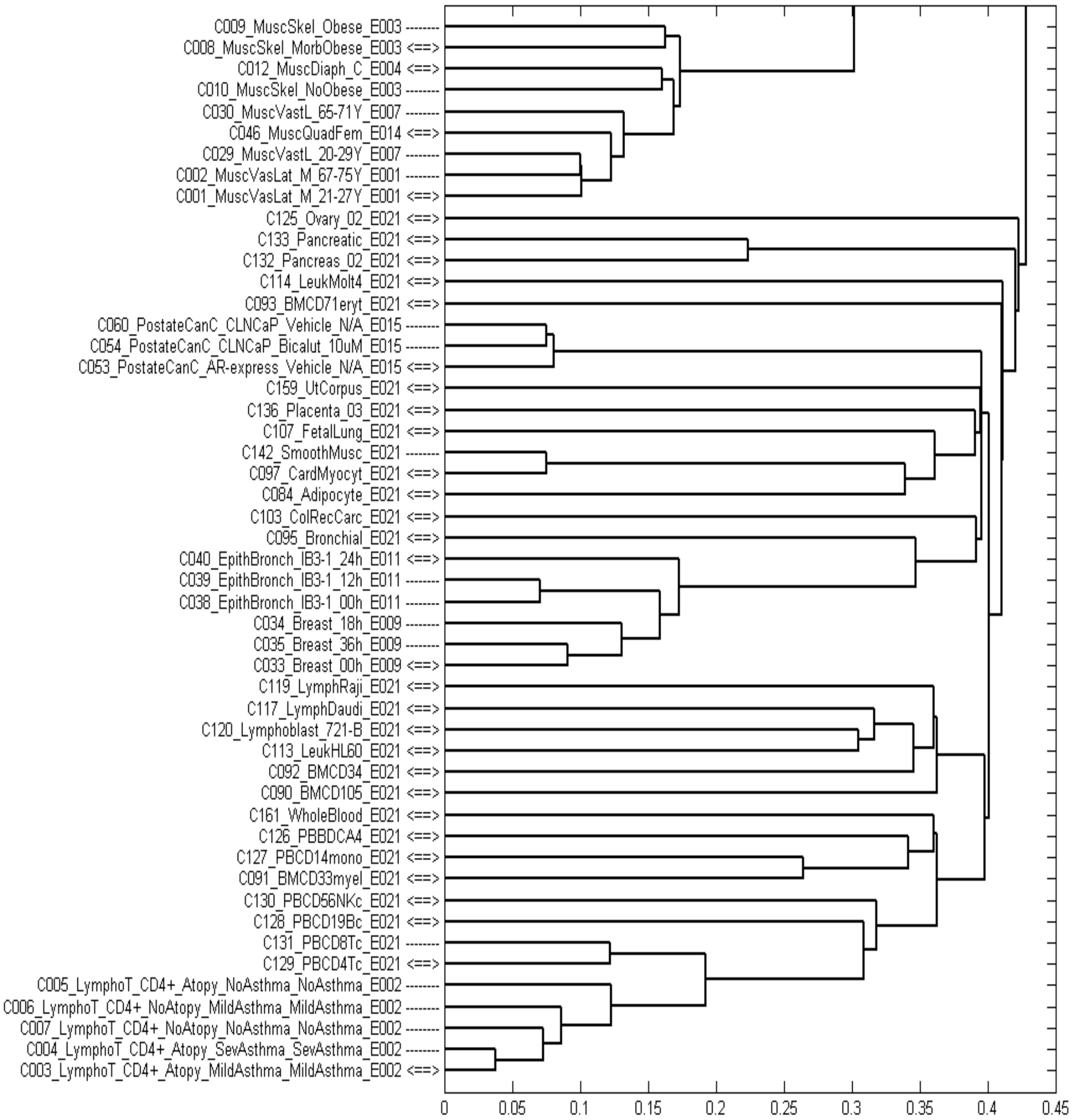

Figure 12 – Portion of the dendrogram of HG U133 biological conditions showing selected non-redundant conditions.

The samples are named as followed: at the beginning is indicated the rank of the biological condition, e.g. C120, followed by a descriptive part constructed as explained in the legend of figure 4, e.g. Lymphoblast_721_B, and finally by the rank of the experiment, e.g. E002. The decision made in view of the distance matrix is indicated after this composite name, using a symbol showing that the biological condition was either used (<==>) or discarded as redundant (----).

References

1. Martin DE, Demougin P, Hall MN, Bellis M: **Rank Difference Analysis of Microarrays (RDAM), a novel approach to statistical analysis of microarray expression profiling data**. *BMC Bioinformatics* 2004, **5**:148.

2. Ghielmetti M, Bellis M, Spycher MO, Miescher S, Vergères G: **Gene expression profiling of the effects of intravenous immunoglobulin in human whole blood**. *Mol. Immunol* 2006, **43**:939-949.

3. Fodor SP, Rava RP, Huang XC, Pease AC, Holmes CP, et coll.: **Multiplexed biochemical assays with biological chips**. *Nature* 1993, **364**:555-556.

4. Nuwaysir EF, Huang W, Albert TJ, Singh J, Nuwaysir K, et coll.: **Gene expression analysis using oligonucleotide arrays produced by maskless photolithography**. *Genome Res* 2002, **12**:1749-1755.

5. **Cardiac Hypertrohy Related to the P13 Kinase Signaling Pathway** [*http://cardiogenomics.med.harvard.edu/groups/proj1/pages/download_pi3k.html*]

6. **Gene Expression Omnibus** [*http://www.ncbi.nlm.nih.gov.geo*]
