# Supplementary figures and images for: Construction and use of gene expression covariation matrix
Source: BMC Bioinformatics. 2009 Jul 13;10:214. doi: 10.1186/1471-2105-10-214 (PMC2720390; doi:10.1186/1471-2105-10-214)

HG-U95 NR1

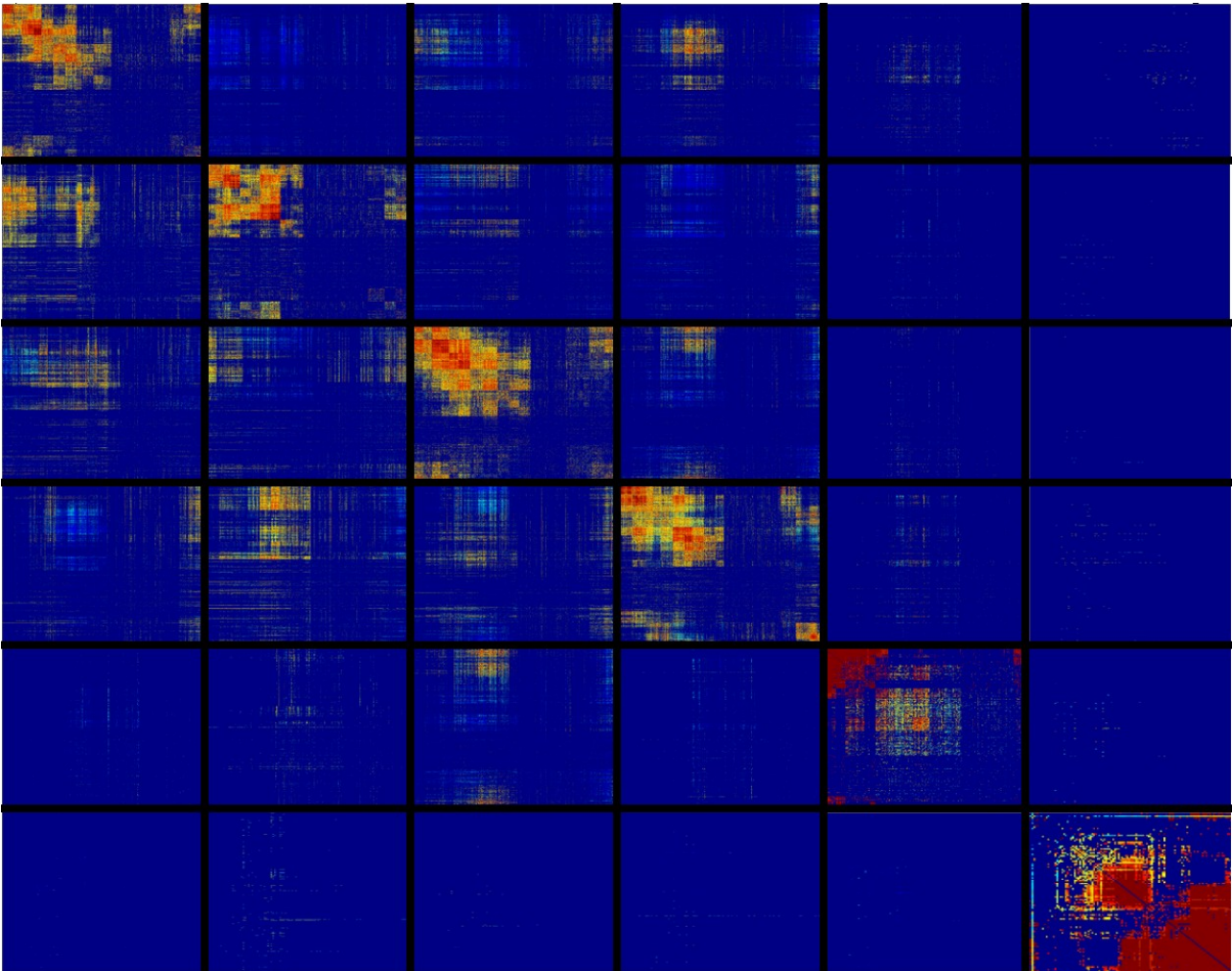

HG-U95 NR10

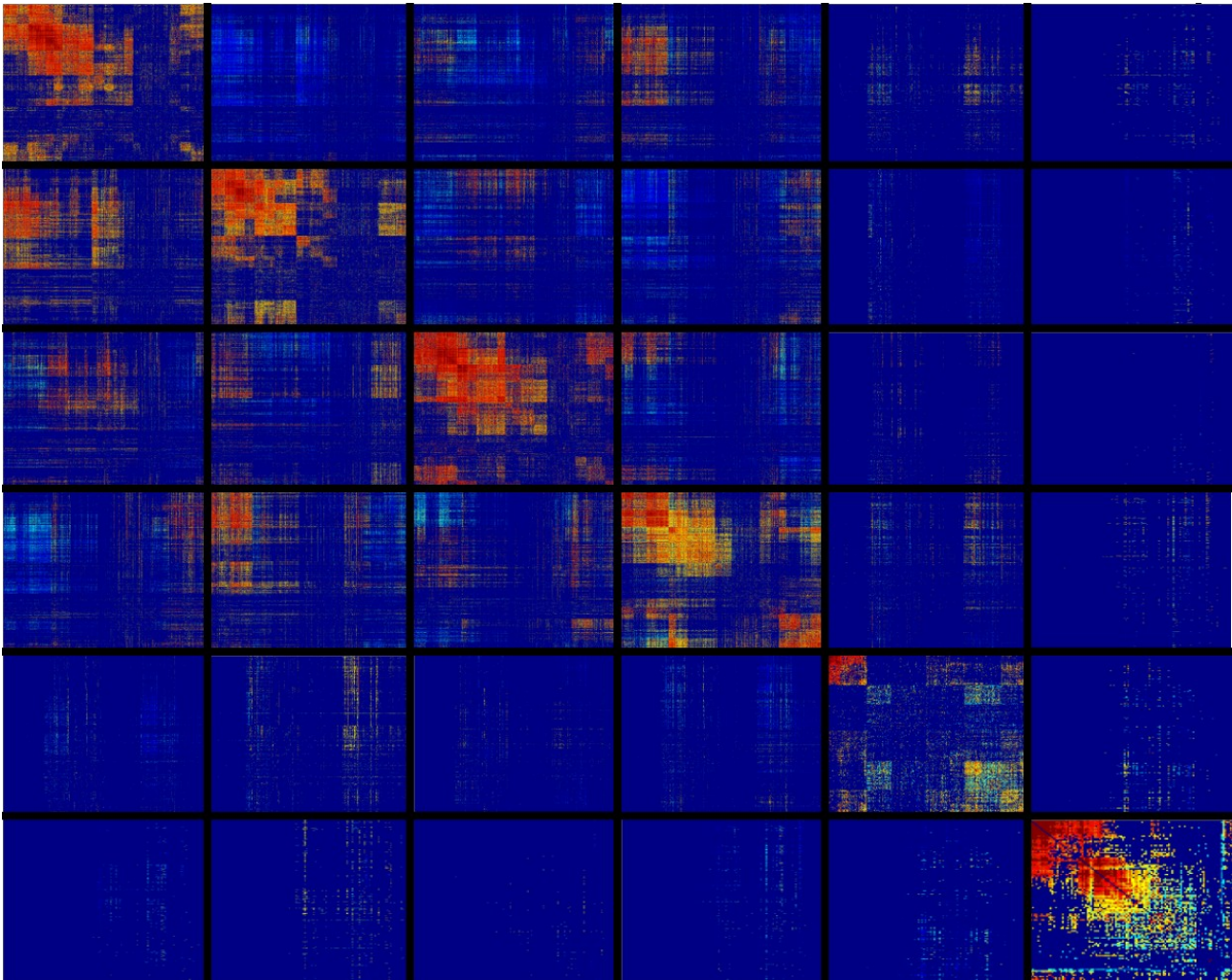

HG-U133 NR1

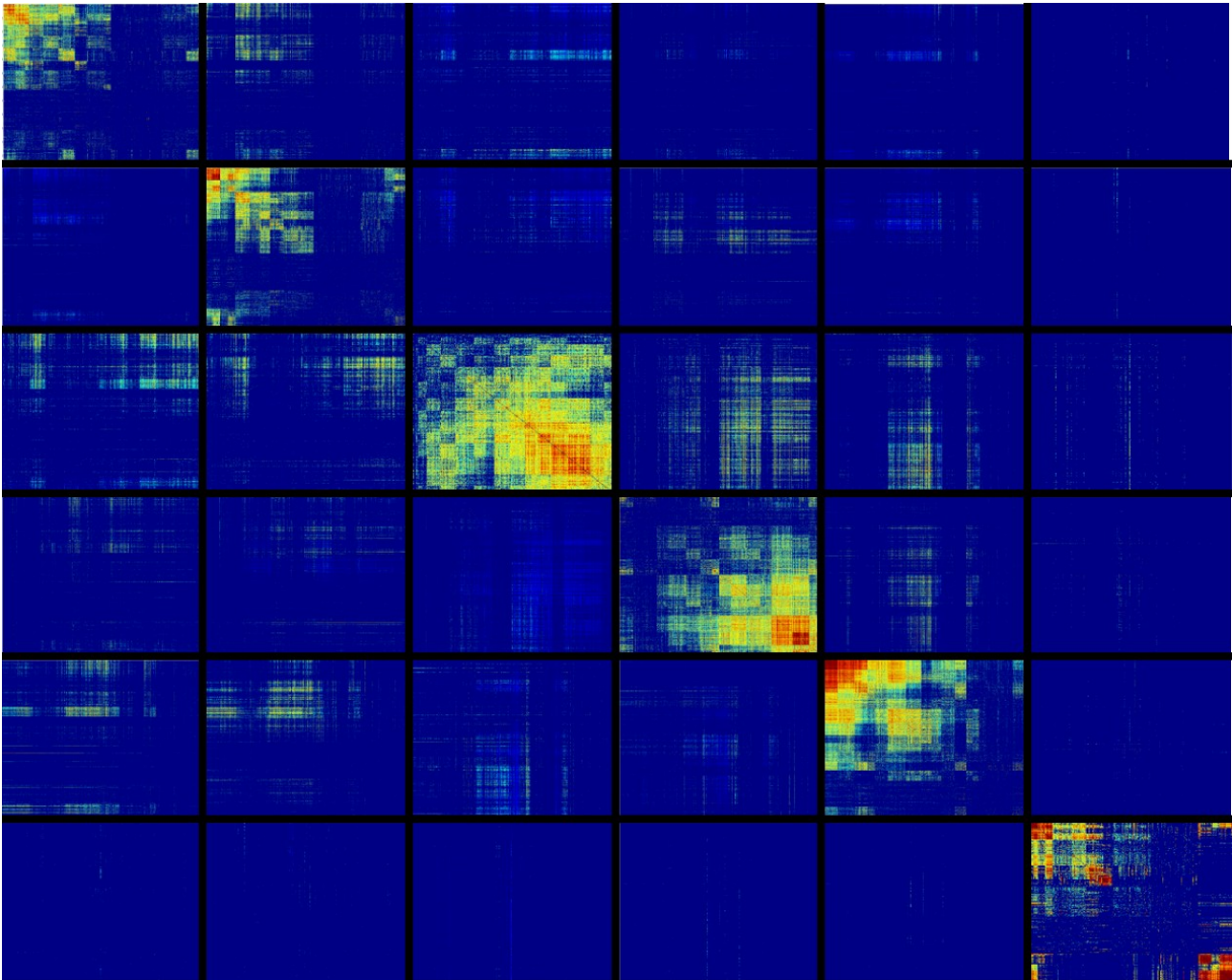

# HG-U133 NR10

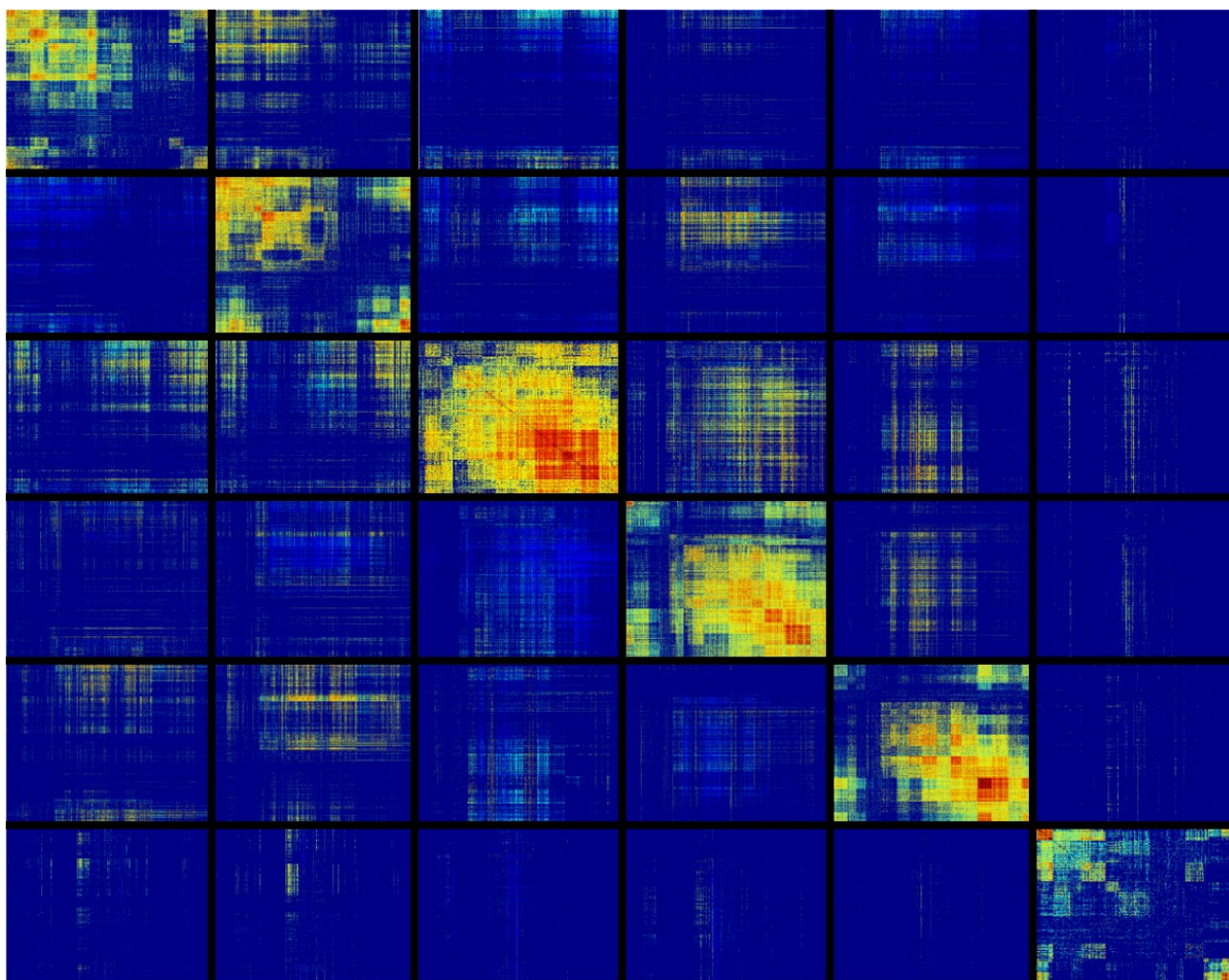

MG-U74 NR1

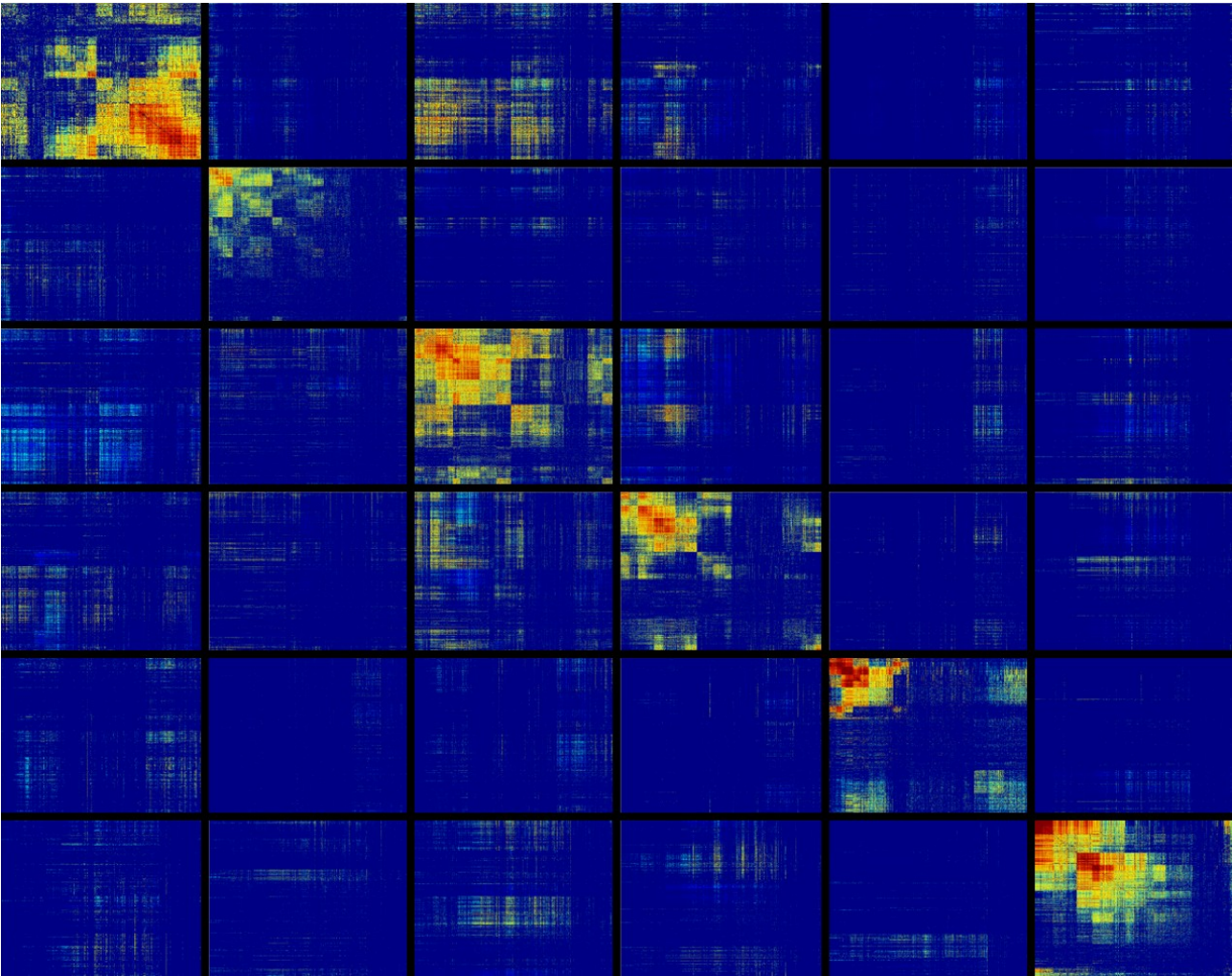

MG-U74 NR10

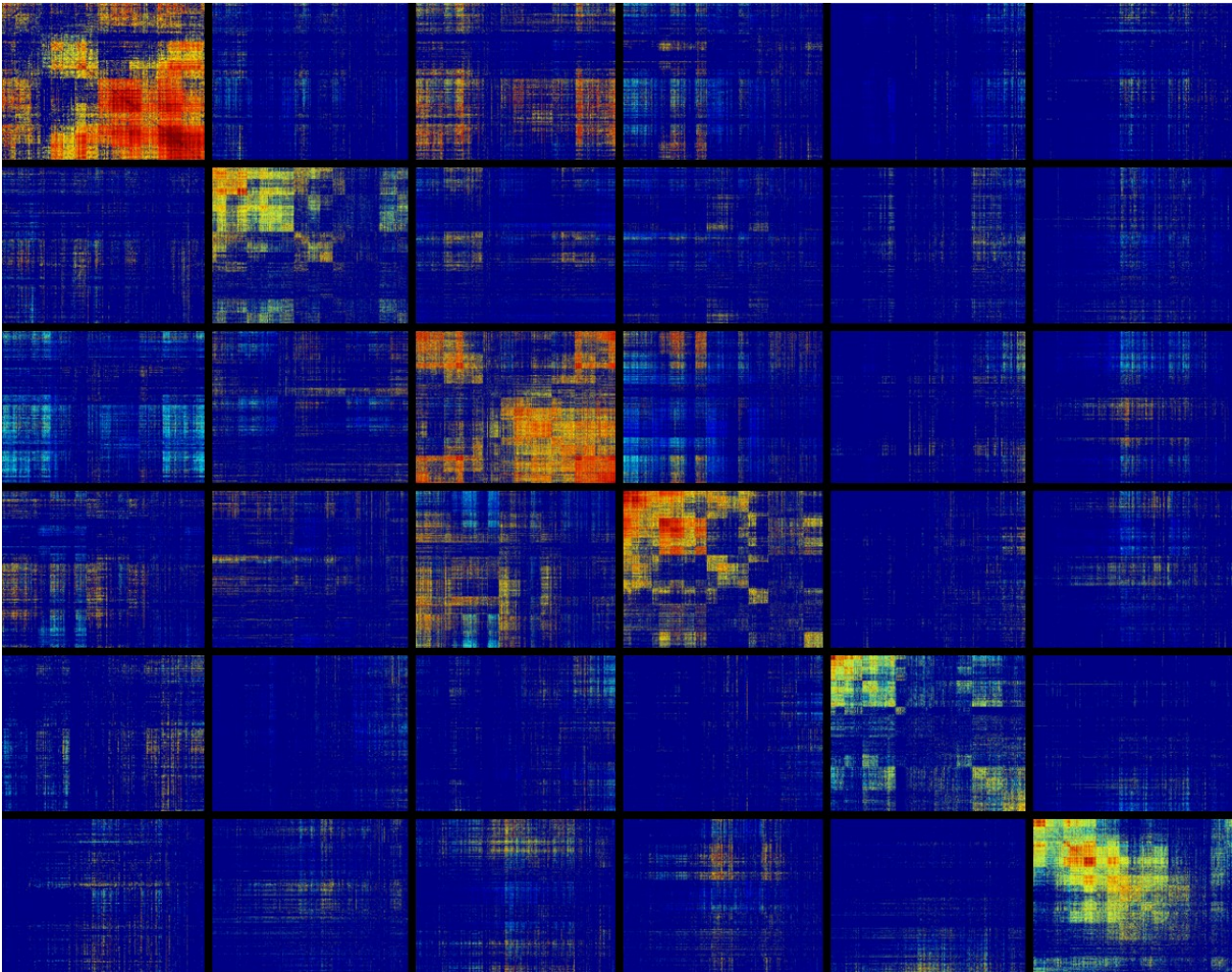

RG-U34 NR1

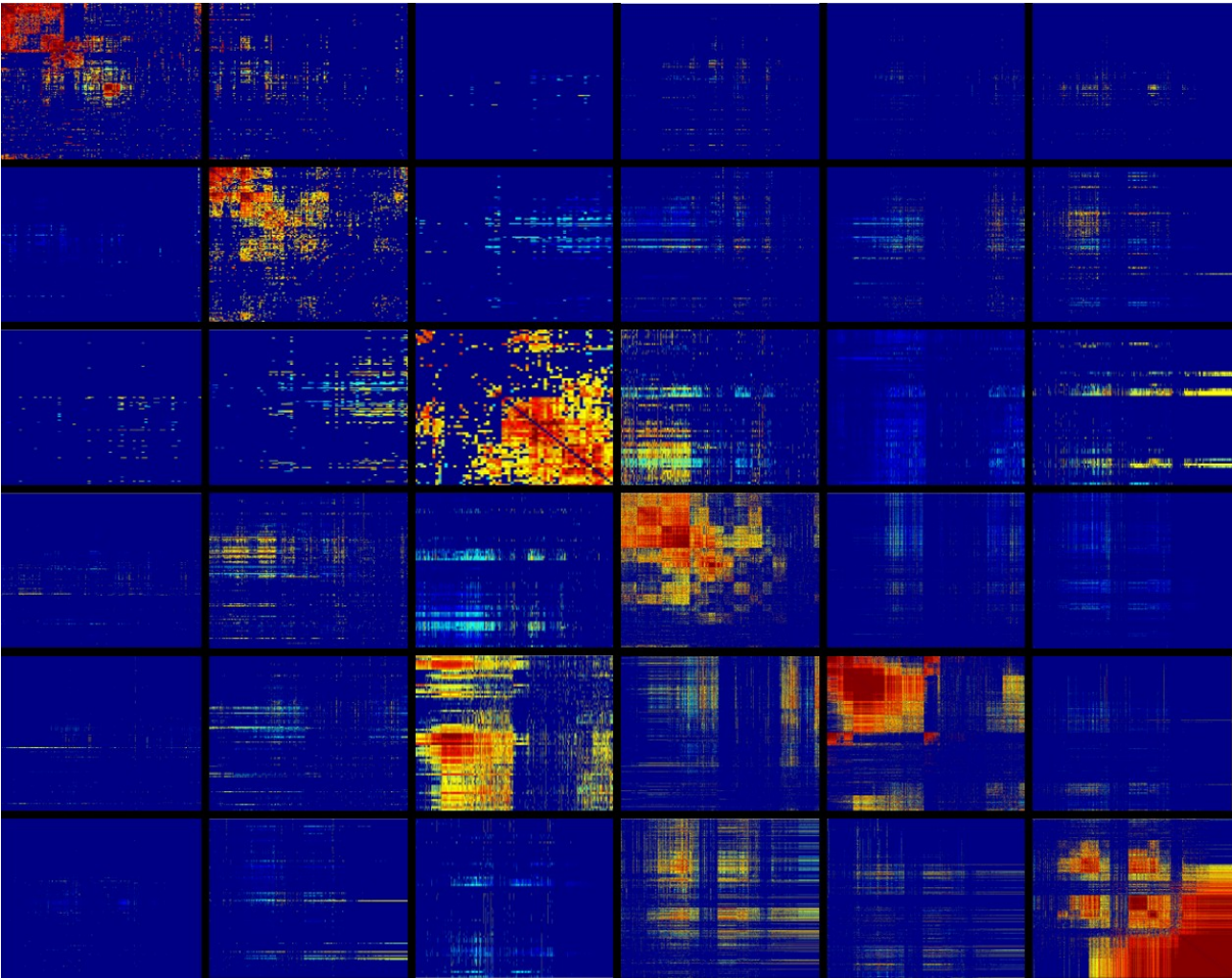

## RG-U34 NR10

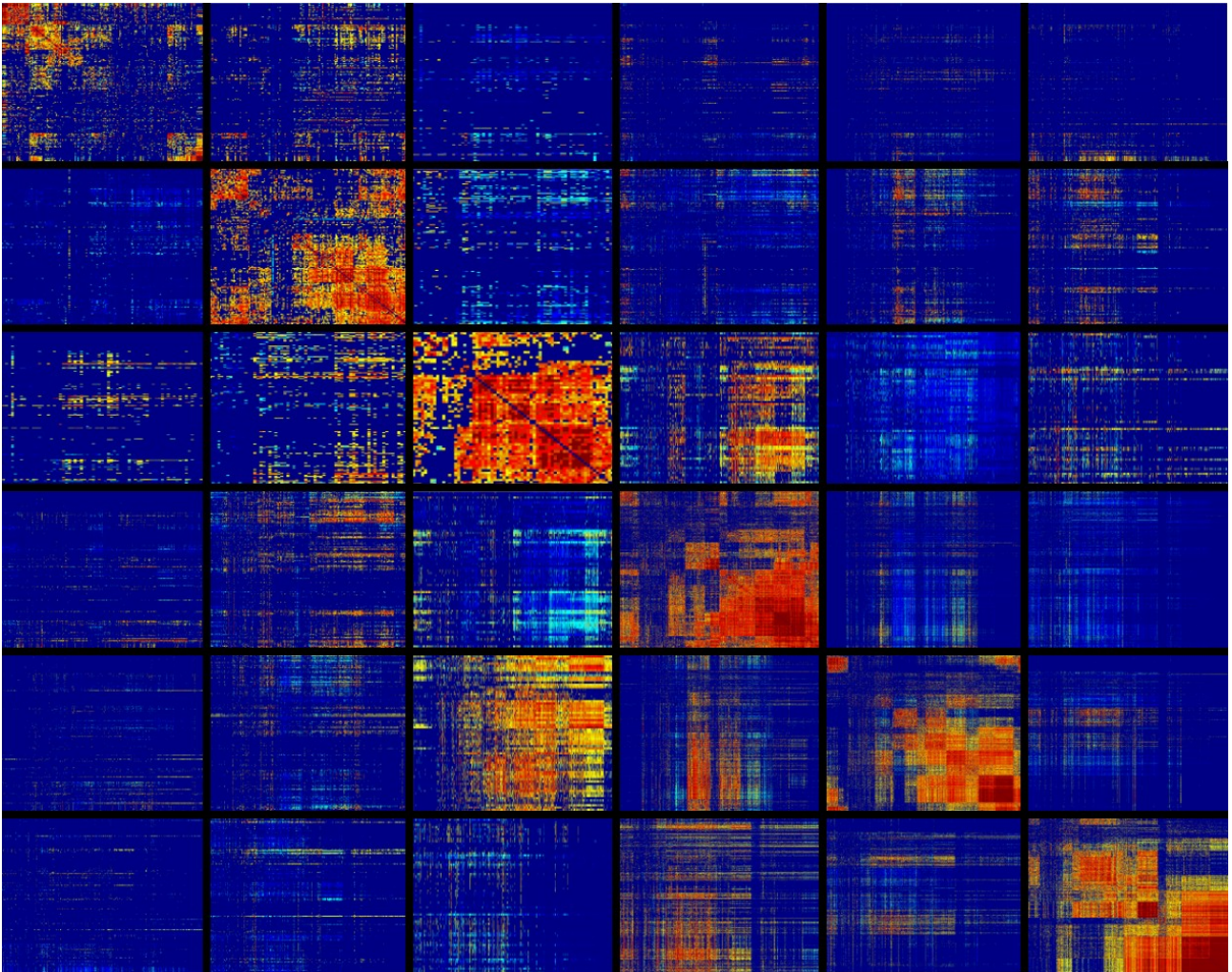

Supplement: Additional file 5 — Images of the CVM. This PDF contains the images of the CVMs with their probe sets ordered by an independent clustering process inside each region, as explained in the legend to Figure 11. [file 1471-2105-10-214-S5.pdf]
